# A conserved MTMR lipid phosphatase increasingly suppresses autophagy in brain neurons during aging

**DOI:** 10.1038/s41598-022-24843-w

**Published:** 2022-12-17

**Authors:** Tibor Kovács, Janka Szinyákovics, Viktor Billes, Gábor Murányi, Virginia B. Varga, Annamária Bjelik, Ádám Légrádi, Melinda Szabó, Sára Sándor, Enikő Kubinyi, Cecília Szekeres-Paracky, Péter Szocsics, János Lőke, Jun Mulder, Balázs Gulyás, Éva Renner, Miklós Palkovits, Károly Gulya, Zsófia Maglóczky, Tibor Vellai

**Affiliations:** 1 Department of Genetics, ELTE Eötvös Loránd University, Pázmány Péter Stny. 1/C, Budapest, 1117 Hungary; 2 MTA-ELTE Genetics Research Group, Budapest, 1117 Hungary; 3 Department of Cell Biology and Molecular Medicine, University of Szeged, Szeged, 6720 Hungary; 4 Department of Ethology, ELTE Eötvös Loránd University, Budapest, 1117 Hungary; 5 Human Brain Research Laboratory, Institute of Experimental Medicine, ELKH, Budapest, 1085 Hungary; 6 Szentágothai János Doctoral School of Neuroscience, Semmelweis University, Budapest, 1085 Hungary; 7 Department of Psychiatry, Saint Borbala Hospital, Tatabánya, 2800 Hungary; 8 Science for Life Laboratory, Department of Neuroscience, Karolinska Institute, 171 77 Stockholm, Sweden; 9 Lee Kong Chian School of Medicine, Nanyang Technological University, Singapore, 636921 Singapore; 10 Human Brain Tissue Bank and Laboratory, Semmelweis University, Budapest, 1085 Hungary; 11 MTA-ELTE Lendület “Momentum” Companion Animal Research Group, Budapest, Hungary

**Keywords:** Genetics, Epigenetics, Gene expression, Autophagy, Neural ageing

## Abstract

Ageing is driven by the progressive, lifelong accumulation of cellular damage. Autophagy (cellular self-eating) functions as a major cell clearance mechanism to degrade such damages, and its capacity declines with age. Despite its physiological and medical significance, it remains largely unknown why autophagy becomes incapable of effectively eliminating harmful cellular materials in many cells at advanced ages. Here we show that age-associated defects in autophagic degradation occur at both the early and late stages of the process. Furthermore, in the fruit fly *Drosophila melanogaster*, the myotubularin-related (MTMR) lipid phosphatase egg-derived tyrosine phosphatase (EDTP) known as an autophagy repressor gradually accumulates in brain neurons during the adult lifespan. The age-related increase in EDTP activity is associated with a growing DNA N*6*-adenine methylation at *EDTP* locus. MTMR14, the human counterpart of EDTP, also tends to accumulate with age in brain neurons. Thus, EDTP, and presumably MTMR14, promotes brain ageing by increasingly suppressing autophagy throughout adulthood. We propose that EDTP and MTMR14 phosphatases operate as endogenous pro-ageing factors setting the rate at which neurons age largely independently of environmental factors, and that autophagy is influenced by DNA N*6*-methyladenine levels in insects.

## Introduction

The accumulation of cellular damage is a characteristic hallmark of essentially all ageing cells^1–7^. Such damages mainly include oxidized, aggregated and misfolded (i.e., non-functional) proteins, which interfere with cellular processes and homeostasis, thereby leading to the senescence and subsequent loss of the affected cells. Massive levels of cell death can then lead to the development of various age-associated degenerative pathologies, particularly neurodegenerative diseases. Thus, the effective elimination of damaged cytosolic materials is crucial for the long-term operation and survival of cells, primarily for those that are terminally differentiated and lost their capacity to proliferate, like neurons.

**Figure 1:**
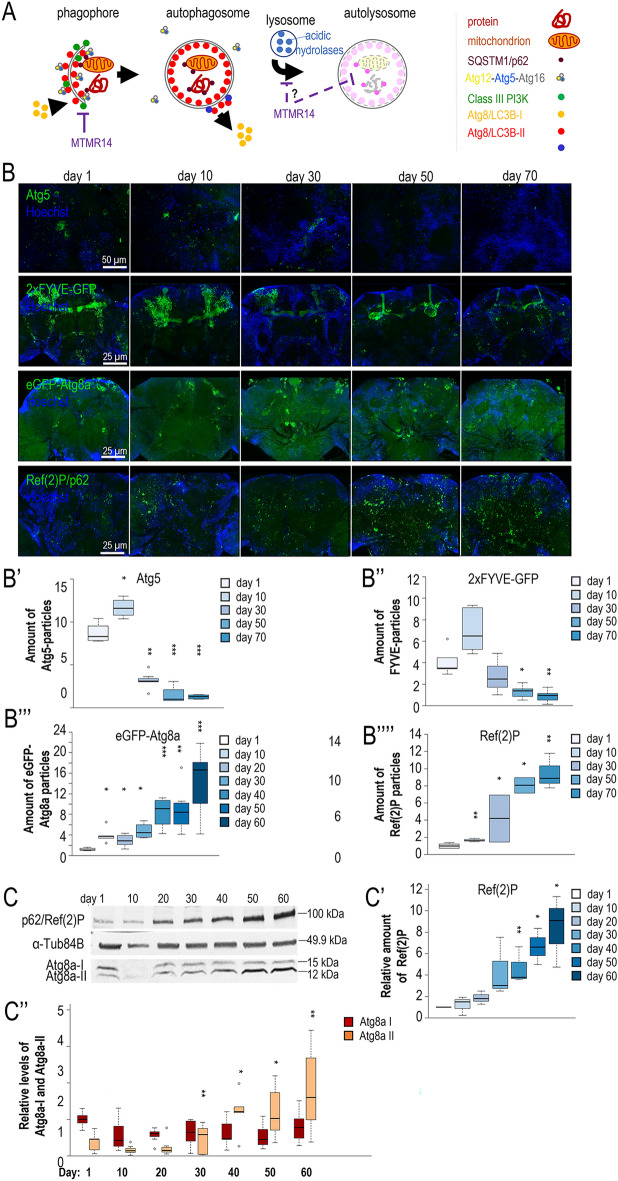
The capacity of autophagy gradually declines with age in the *Drosophila* brain. (**A**) The mammalian macroautophagic process. During autophagy, unwanted cytoplasmic constituents (proteins and mitochondria are indicated) are sequestered into a double membrane-bound vesicle called autophagosome. Autophagosome is formed by the elongation and fusion of a phagophore membrane. The scheme indicates where Atg5, class III PI3K, Atg8/LC3B-II and SQSTM1/p62 autophagic markers exert their effects during the process. Atg5 and PI3K (the latter is indicated by 2xFYVE-GFP) label early stages of the process (phagophore formation), Atg8/LC3B-II designates both phagophores and autophagosomes, while p62 is an adaptor protein serving as a substrate for autophagic breakdown. Atg8-I: soluble form; Atg8-II: membrane-conjugated form. The cysteine protease Atg4 deconjugates Atg8-II from the autophagosomal membrane (i.e., it mediates the conversion of Atg8-II to Atg8-I) when autophagosome is formed. MTMR14 inhibits autophagic membrane formation by antagonizing the class III PI3K complex. Bars indicate negative regulatory interactions, arrows indicate activations. (**B**) Levels of Atg5- (first row), 2xFYVE-GFP- (second row), eGFP-Atg8a (third row) and Ref(2)P/p62-positive structures (forth row) in the brain of *Drosophila* adults at different ages. Atg5 and 2xFYVE-GFP label early autophagic structures, phagophores and nascent autophagosomes. In each row, fluorescence microscopic images were captured with the same exposure time. For 2xFYVE-GFP, a GFP-tagged transgene was used, otherwise specific antibodies were used. Scale bars correspond to 25 µm. Hoechst staining (blue) indicates nuclei. (**B′–B″″)** Quantification of Atg5-, 2xFYVE-GFP-, eGFP-Atg8a- and Ref(2)P-positive structures. (**C**) Western blot analysis showing relative Ref(2)P and Atg8a-I/II levels in whole head extracts dissected at different adult stages. Atg8a-II labels phagophores and autophagosomes. α-Tub84B was used as an internal control. (**C,C″**) Quantification of relative Ref(2)P densities, as well as relative Atg8-I and Atg8-II levels determined by the Western blot analysis (**C**). In panels (**B′**–**B″″**), (**C′**) and (**C″**), on the plot the boxes represent the most typical 50% of the samples, the line indicates the median, upper and lower whiskers show remaining 25–25% of the samples. Circles mark outliers. *P < 0.05; **P < 0.01; ***P < 0.001 at each comparison with day 1. For statistics, see the “Materials and methods”.

Autophagy acts as a major catabolic process of eukaryotic cells by which cellular damage can be effectively eliminated^8–12^. During autophagy, parts of the cytoplasm are delivered into lysosomes for degradation by acidic hydrolases. Depending on the mechanism by which autophagic cargo is delivered into the lysosomal compartment, three major types of autophagy can be distinguished: microautophagy, chaperone-mediated autophagy and macroautophagy. Macroautophagy (hereafter referred to as autophagy) involves the formation of a double membrane-bound vesicle called autophagosome to sequester the cytoplasmic materials destined for degradation. The autophagosome then fuses with a lysosome to form an autolysosome, in which the enzymatic breakdown eventually takes place (Fig. 1, A). Defects in the autophagic process are implicated in the development of diverse neurodegenerative pathologies^9,13,14^. This raises the possibility that autophagy works less effectively in neurons at advanced ages as compared to early adult stages. In the nematode *Caenorhabditis elegans* and fruit fly *Drosophila melanogaster*, autophagy was indeed found to operate at significantly lower levels in aged animals than in young adults^15–17^. This age-related decline in autophagic capacity is accompanied by a decreased expression of a key autophagy-related (*Atg*) gene, *Atg8/LC3B* (microtubule-associated proteins 1A/1B light chain 3B), which encodes a ubiquitin-like protein required for the formation of autophagic membrane structures^18^. Despite its physiological and medical significance, it is still largely unknown why the capacity of autophagy declines with age in neurons. Stochastic processes including random inactivating mutations in *Atg* genes in the genome of individual neurons should certainly contribute to the decay^6,7^. Regulatory factors yet largely unexplored may also be involved. According to a recent study, Rubicon (RUN domain and cysteine-rich domain containing, Beclin 1-interacting protein), which inhibits autophagy through interacting with a protein complex containing Beclin 1 (coiled-coil, myosin-like BCL2-interacting protein), Vps15/p150 (Vacuolar protein sorting 15), PI3K (the class III phosphatidylinositol-3 kinase) and UVRAG (ultraviolet irradiation resistance-associated gene) increasingly downregulates the process during ageing in worms, flies and mice^19^. However, why Rubicon progressively accumulates with age in various cell types remains unresolved.

The formation of early autophagic membrane structures requires certain phosphoinositide (PI) derivatives such as phosphatidylinositol 3-phosphate (PI3P), which is converted from PI by class III PI3K enzyme (Fig. 1A)^20^. PI3K, also called Vps34 (vacuole protein sorting), is a member of the autophagic vesicle nucleation complex. Under normal conditions, the mammalian myotubularin-related lipid phosphatase MTMR14 and its *Drosophila* orthologue EDTP (Egg-derived tyrosine phosphatase) antagonize PI3K/Vps34 to prevent the harmful hyperactivation of autophagy^21–23^. MTMR14 inhibits basal autophagy through converting PI3P into PI^24^. In genetic backgrounds defective for MTMR14 or EDTP, the amount of PI3P-enriched structures become elevated relative to control^22,25^. Beside this initial stage of autophagy, MTMR14 also regulates a later stage of the process, at the fusion of autophagosome with a lysosome (Fig. 1A)^22^. In this study we show that EDTP and MTMR14 increasingly accumulate with age in brain neurons. These conserved MTMR lipid phosphatases contribute to the age-dependent decline of autophagy in neurons, thereby promoting brain ageing.

## Results

### Both early and late stages of autophagy become impaired in brain neurons during ageing

To understand better how autophagy declines with age, we first determined relative levels of the autophagic activity during the adult lifespan in *Drosophila*. Two widely used markers for monitoring early stages of autophagy, Atg5 and the 2xFYVE domain (Fig. 1A)^26,27^, displayed gradually decreasing accumulation levels in brains isolated from adult flies at different life stages (Fig. 1B–B″). Because Atg5 plays a role in the extension of the growing isolation membrane called phagophore, its level correlates to the amount of the structure^28^. The FYVE domain binds PI3P, hence its quantity is proportional to PI3K/Vps34 activity^29^. The application of these markers clearly demonstrated that phagophore formation gradually declines in brain neurons as the organism ages. We also assessed the amount of the autophagic membrane-conjugated form of Atg8a, Atg8a-II^30^. The amount of Atg8a-positive autophagic structures labelled by an endogenously expressed eGFP-Atg8a reporter^31^ became progressively elevated in the brain during the adult lifespan (Fig. 1B–B ‴). Because eGFP is sensitive to low pH, it is inactive in acidic compartments, thereby labelling phagophores and autophagosomes, but not autolysosomes. Similar results were obtained when testing Atg8a-II levels in brain extracts derived from adult animals at different stages, using a western blot analysis (Fig. 1C,C″). Contrary to Atg8a-II, the level of the non-conjugated, soluble form of Atg8a (Atg8a-I) remained nearly constant throughout adulthood. These data indicate that, despite lowered phagophore formation, autophagosomes were generated at a progressively increasing rate during the adult lifespan. Alternatively, a later stage of the degradation process was also affected, leading to a net accumulation of autophagosomes or non-digestive autolysosomes. Possibly autophagosome-lysosome fusion, lysosomal acidification or degradation of autolysosomal content is the stage impacted.

To distinguish between the two alternatives above, we assessed the amount of Ref(2)P, the fly counterpart of human p62/SQSTM1 (Sequestosome 1) during the adult lifespan^15^. Because p62/SQSTM1 serves as a substrate for autophagic degradation (the protein links the cargo to membrane-bound Atg8/LC3B), its level is inversely proportional to autophagic activity^32,33^. Using a Ref(2)P-specific antibody, we performed an immunohistochemical analysis on brain samples dissected at different adult stages, and found that the older the animal, the higher the amount of insoluble protein aggregates labelled by the antibody (Fig. 1B,B″″). To strengthen these results, a subsequent western blot analysis was applied to whole head samples, using the same Ref(2)P-specific antibody. Consistent with data obtained by fluorescence microscopy, the amount of soluble Ref(2)P protein became gradually elevated with age in head extracts (Fig. 1C,C′). Together, these results imply that during ageing autophagic degradation becomes impaired at two stages of the process. First, as revealed by lowered Atg5 and PI3P levels, at vesicle nucleation when the phagophore forms and grows. Second, as indicated by increased Atg8a-II accumulation, after autophagosome formation when the structure fuses with a lysosome or the autolysosomal content is digested enzymatically. We conclude that autophagy gradually declines with age in brain neurons due to the cumulative effect of suppressed autophagosome formation and compromised autolysosomal function.

### EDTP progressively accumulates in brain neurons during ageing

**Figure 2:**
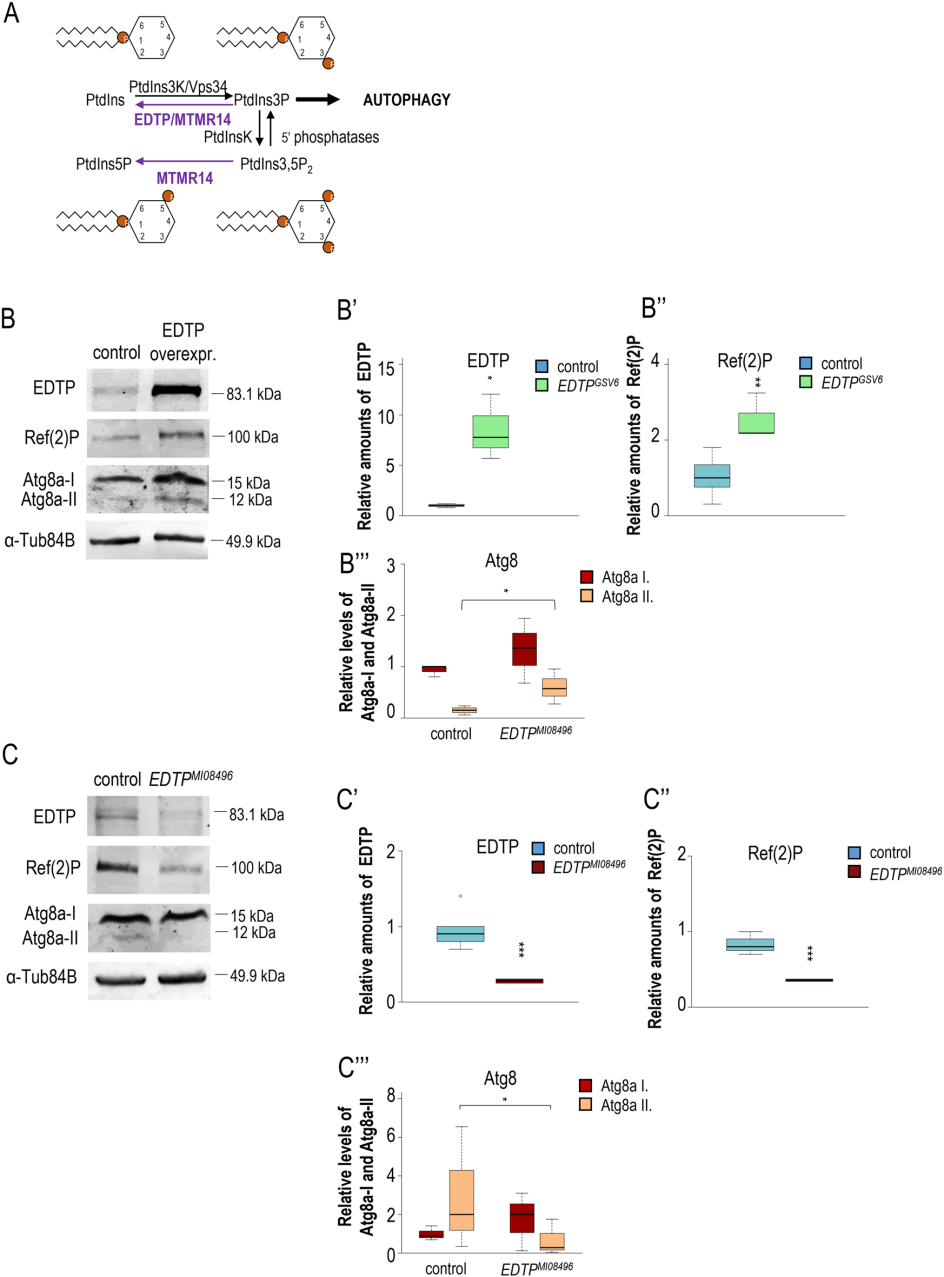
EDTP hyperactivity inhibits, while EDTP deficiency enhances, autophagic activity in the *Drosophila* brain. (**A**) Enzymatic function of *Drosophila* EDTP and mammalian MTMR14 lipid phosphatases. The two proteins convert PI3P into PI, thereby antagonizing autophagic membrane formation. (**B**) Western blot analysis demonstrates elevated levels of EDTP and Ref(2)P in an genetic background overexpressing *EDTP* as compared to control. The autophagic membrane-conjugated form of Atg8a (Atg8a-II) also increases relative to Atg8a-I, which represents the soluble (non-conjugated) form of Atg8a. (**B′–B‴)** Quantification of relative EDTP, Ref(2)P, Atg8a-I and Atg8a-II levels, determined by the western blot analysis (**B**). (**C**) Western blot analysis reveals that relative levels of EDTP, Ref(2)P and Atg8a-II/Atg8a-I ratio each decrease in an EDTP defective (hypomorphic mutant) genetic background relative to control. (**C′–C‴**) Quantification of relative EDTP, Ref(2)P, Atg8a-I and Atg8a-II densities identified by the western blot analysis (**C**)**.** In panels B and C, proteins were extracted from the head of female fruit flies, αTub84B was used as an internal control, and animals were maintained at 29 °C. On the plot the boxes represent the most typical 50% of the samples, the line indicates the median, upper and lower whiskers show remaining 25–25% of the samples. Circles mark outliers. *P < 0.05; **P < 0.01; ***P < 0.001. For statistics, see the “Materials and methods”.

MTMR14 has been shown to influence autophagy at both early (phagophore formation) and late (autolysosome formation) stages of the process (Figs. 1A, 2A)^22^. Furthermore, we demonstrated previously that EDTP effectively inhibits basal autophagy in the *Drosophila* fat body^23,25,34^, and that MTMR14 abundantly accumulates in the human brain cortex^35^. Here, we revealed that Atg8a-II and Ref(2)P levels increase in an *EDTP*-overexpressing genetic background (Fig. 2B–B‴), but lower in an *EDTP* hypomorphic mutant background (Fig. 2C–C‴). Furthermore, *EDTP* overexpression significantly elevated the amount of ubiquitinated structures (Fig. S1A-A′). This suggests that under normal conditions EDTP also inhibits autophagy, besides at autophagosome formation, following Atg8a lipidation.

**Figure 3:**
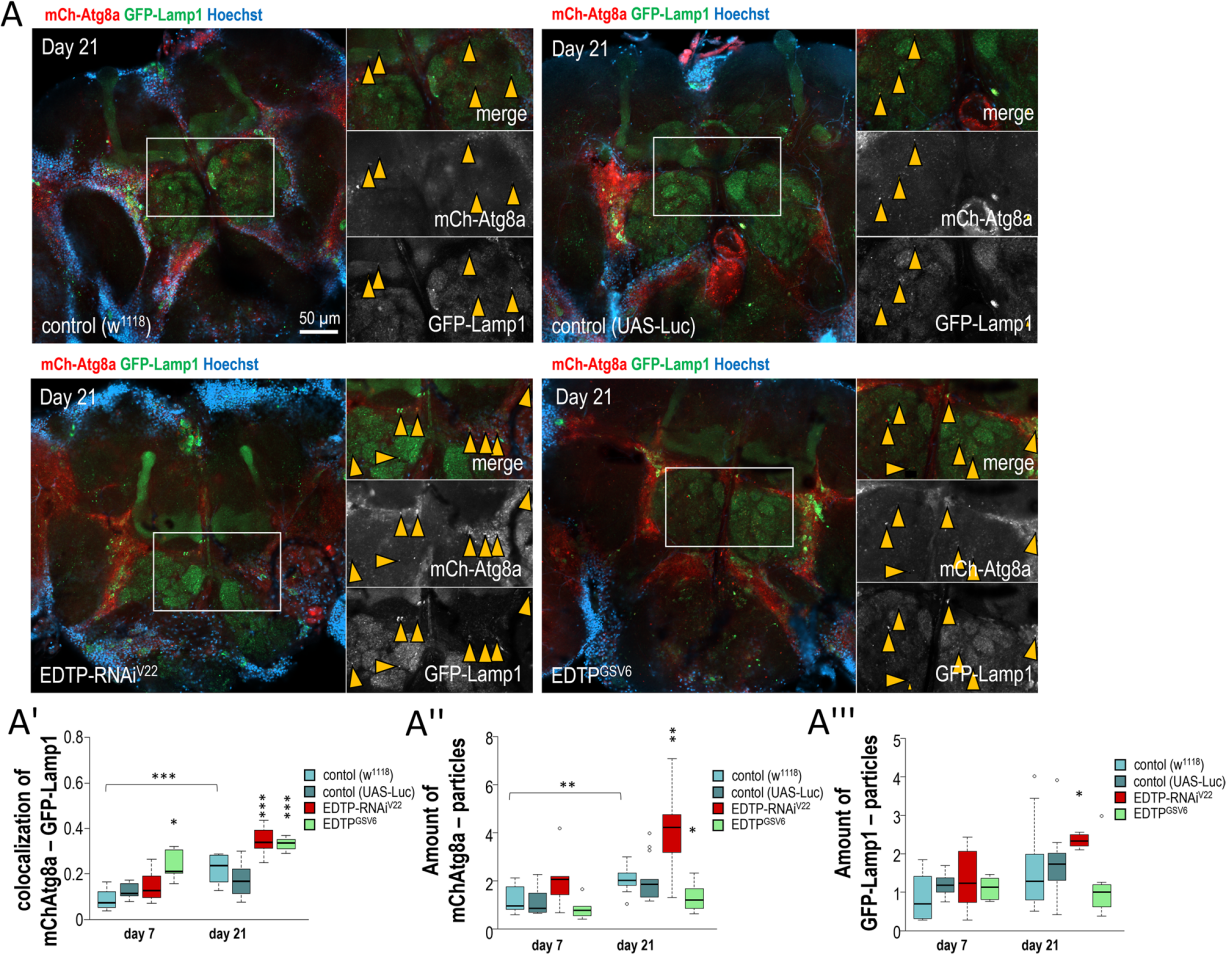
EDTP inhibits both autophagic vesicle nucleation and acidic breakdown in the *Drosophila* brain. (**A**) Fluorescent images showing co-localisation of GFP-Lamp1 (green) and 3xmCherry-Atg8a (red) reporters in neurons of the adult *Drosophila* brain. Yellow arrows indicate structures labelled by both markers. White squares indicate the enlarged areas. On the diagrams, statistical data of samples derived from animals at the adult stages of 7 and 21 days are shown (**A′–A‴**). Animals were maintained at 29 °C. On the plot, the boxes represent the most typical 50% of the samples, the line indicates the median, upper and lower whiskers show remaining 25–25% of the samples. Circles mark outliers. *P* < 0.05; **P < 0.01; ***P < 0.001. For statistics, see the “Materials and methods”.

To understand better the neuronal roles of EDTP in autophagy control, we monitored the co-localisation of mCherry-Atg8a and GFP-Lamp1 (lysosomal structure-specific) reporters in 7- and 21-day-old adults maintained at 29 °C (Fig. 3A,A′ and Fig. S1B–B‴). *EDTP* silencing (*EDTP-RNAi*^*V22*^) and overexpression (*EDTP*^*GSV6*^) each increased the co-localisation of these markers in aged animals relative to control at the same age. In control genotypes, the amount of structures labelled by both markers was significantly higher in aged adults than in young ones (Fig. 3A″–A‴ and Fig. S1B′). *EDTP* downregulation increased, while EDTP hyperactivity lowered, the number of mCherry-Atg8a- and GFP-Lamp1-specific structures (Fig. 3A″,A‴ and Fig. S1B′). The lipidated form of Atg8a (Atg8a-II) is a substrate of autophagic degradation, and the mCherry reporter tolerates the acidic milieu of autolysosomes^36,37^. This can explain why EDTP deficiency elevates the amount of autolysosomes (mCherry-Atg8a-positive structures) in a fluorescent microscopic assay but decreases Atg8a-II levels in a western blot analysis (Fig. 3A,A″ and Fig. 2C,C‴). Thus, *EDTP* overexpression might inhibit autophagy by generating less autophagic structures. Although these structures are capable of fusing with lysosomes, the breakdown process appears to be compromised (as indicated by accumulating Atg8a-II levels and increased mCherry-Atg8a—GFP-Lamp1 co-localisation) (Figs. 2, 3). MTMR proteins are known to dephosphorylate lipids involved in autophagy, such as PI(3,5)P2^38^, which is required for downstream stages of the autophagic process, including acidification of lysosomes^39^, and lysosomal biogenesis^40^. According to our results, EDTP may simultaneously block vesicle nucleation (via antagonizing Vsp34 complex) and lysosomal degradation in neurons.

**Figure 4:**
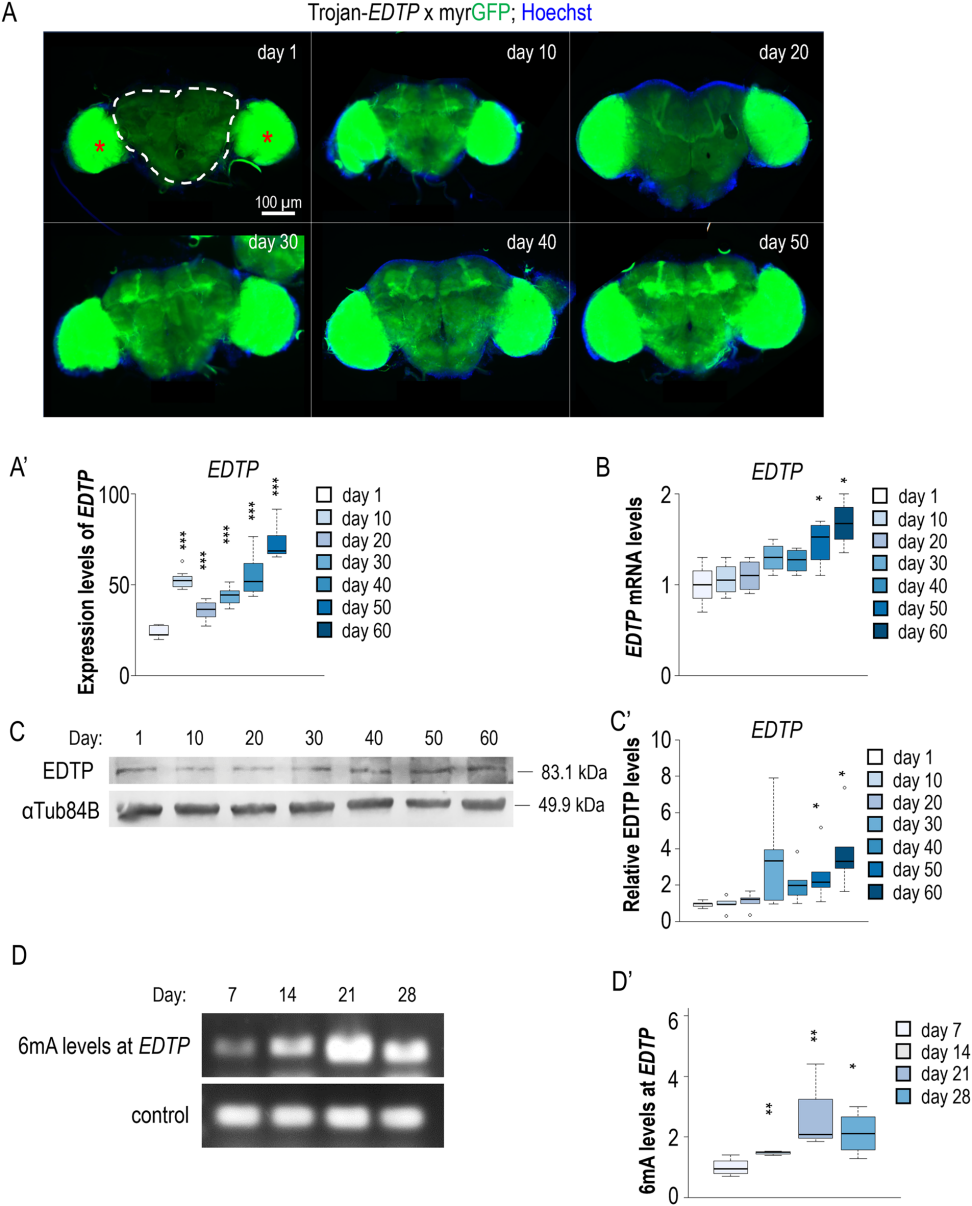
*EDTP* is increasingly expressed in brain structures during the *Drosophila* adult lifespan. (**A**) Fluorescence microscopic images showing the expression of an *EDTP*-trojan gene (transcriptional activity of *EDTP*) trap system in the brain dissected at different stages of adulthood (days are indicated). Images were captured with the same exposure time. Hoechst staining (blue) indicates nuclei. Red asterisks indicate the medullas (intense glowing) that were excluded from the analysis. Scale bar corresponds to 100 µm. A white dotted line outlines the brain section where the analysis was performed. (**A′**) Quantification of relative *EDTP* expression levels in the brain of adult flies at different ages. (**B**) qPCR analysis on brain extracts shows that *EDTP* transcript levels are higher in aged (day 50 and day 60) than in young (day 10) adults. (**C**) Western blot analysis reveals that EDTP tends to accumulate with age in *Drosophila* head extracts. αTub84B was used as an internal control. (**C′**) Quantification of relative EDTP levels in head extracts at different adult stages, determined by the western blot analysis (**C**). Animals were maintained at 25 °C. In panels (**A′**)), (**B**) and (**C′**)), the boxes represent the most typical 50% of the samples, the line indicates the median, upper and lower whiskers show remaining 25%-25% of the samples. Circles mark outliers. *P < 0.05, **P < 0.01, ***P < 0.001 at each comparison with day 1. For statistics, see the “Materials and methods” and Table S1. (**D**) *N*^*6*^-adenine methylation at the *EDTP* locus increases gradually with age in *Drosophila.* Relative *N*^*6*^-methyladenin (6 mA) levels at *EDTP* locus at different adult stages. (**D′**) Quantification of relative 6 mA levels at *EDTP* site. Animals were maintained at 29 °C. In panels (**D′**), *P < 0.05, **P < 0.01 at each comparison with day 7.

Relevant literature data and results above prompted us to examine adult stage-associated relative levels (i.e., accumulation dynamics) of these conserved MTMR lipid phosphatases in brain neurons throughout the adult lifespan. To this end, we assayed EDTP accumulation in the brain throughout the adult lifespan. *EDTP* expression was first monitored by a fluorescent gene trap system, in which a Trojan *EDTP-Gal4* driver is inserted into the first intronic sequence of *EDTP* gene, controlling *UAS-myr-GFP* reporter activity (Fig. S2A). *EDTP* activity exhibited a gradual, age-associated increase in the organ (Fig. 4A,A′). Nearly a threefold difference was detected between young (day 10) and aged (day 60) adults. This age-related shift in *EDTP* transcription was particularly evident in brain structures called mushroom bodies, subesophageal ganglions and antennal lobes (Fig. 3A and Fig. S1B), and was not accompanied by an increase in the number of neurons accumulating EDTP (Fig. S2C,C′). During ageing, the levels of autophagic markers significantly changed in the area of mushroom body; the amount of structures labelled by 2xFYVE-GFP became lowered whereas Ref(2)P levels increased (Fig. S2D–E′). A quantitative PCR analysis on head samples also displayed higher amounts of *EDTP* transcripts in old animals relative to young ones (Fig. 4B). These results reveal that *EDTP* transcript levels gradually increase in brain neurons as the animal ages, which is in line with a previous genome-wide gene expression analysis identifying genetic factors that are up- or downregulated during *Drosophila* ageing^41^.

Using an EDTP-specific antibody, we next tested the amount of the protein in whole head extracts. The antibody was capable of labelling EDTP in the wild type, but largely failed to mark an EDTP-positive band in the *EDTP*^*MI08496*^ mutant background (Fig. 2C–C‴). A conducted western blot analysis uncovered that EDTP tends to increasingly accumulate in the head during the adult lifespan (Fig. 4C–C′). Thus, EDTP activity progressively increases with age in the *Drosophila* brain.

To understand why *EDTP* expression increases with age in brain neurons, we examined changes in N*6*-methyladenine (6 mA) DNA modification at *EDTP* locus throughout adulthood (in general, methylation of adenine on the N*6* position promotes transcription at the affected locus). Relative 6 mA levels at any genomic site containing a GATC sequence can be assessed by a PCR-based method, which involves a methylation-sensitive DpnI enzymatic digestion of the genomic DNA and a PCR amplification of the target site (Fig. 4D)^42^. We found that relative 6 mA levels at the target site tend to elevate during the adult lifespan, a drop in 6 mA levels could be seen only at the latest adult stages (Fig. 4D–D′). Thus, age-related changes in *EDTP* expression and, as a consequence, changes in autophagic activity, may be epigenetically determined in this organism. Interestingly, a similar change was not detectable in case of human *MTMR14* gene (data not shown).

### *EDTP* downregulation in dopaminergic neurons delays the incidence of age-associated neuronal dysfunctions

**Figure 5:**
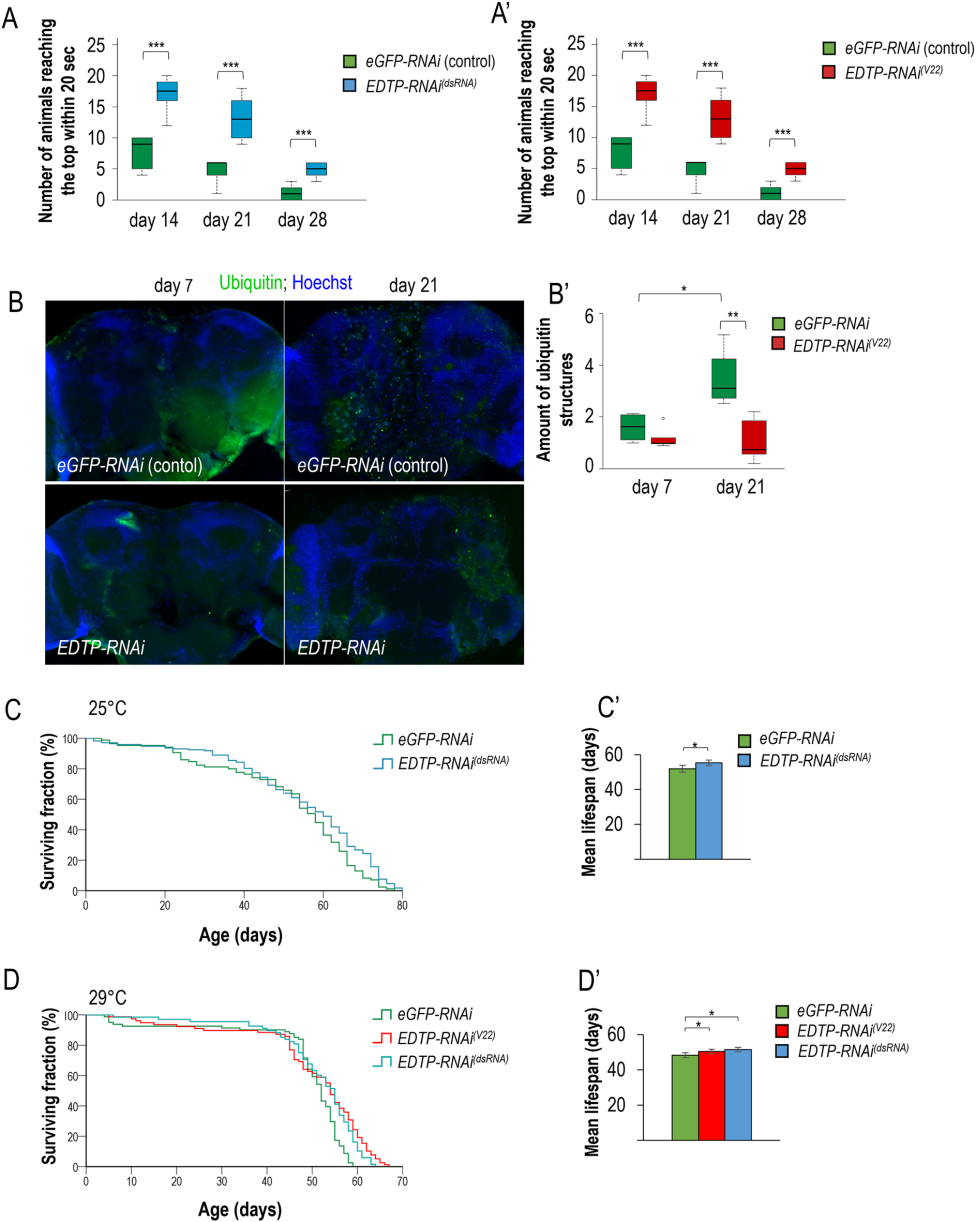
Downregulation of EDTP in neurons can improve climbing ability, lower protein ubiquitination in the brain, and extend lifespan. (**A–A′**) Using two different RNAi constructs (also see Fig. S2B,B′), *EDTP* was downregulation in dopaminergic neurons. Flies were maintained at 29 °C, *eGFP-RNAi* (indicates *ON target*-free *UAS* transgene control) and *EDTP-RNAi* were driven by a *ple-Gal4* driver expressed in dopaminergic neurons only. (**B**) Fluorescent images showing the accumulation of ubiquitinated proteins (green aggregates) in the brain of adult flies at different stages (7 and 21 days). *eGFP-RNAi* was used as control. Hoechst staining (blue) indicates nuclei. Animals were maintained at 29 °C. Scale bar represents 40 µm. RNAi constructs were driven by *Appl-Gal4*. (**B′**) Quantification of ubiquitinated proteins at two different adult stages. (**C**) Kaplan-Meyer lifespan curves of *eGFP-RNAi* (ON-target free *RNAi* control) versus *EDTP-RNAi*^*(dsRNA)*^ (*EDTP* was downregulated in dopaminergic neurons specifically) flies. Animals were maintained at 25 °C. (**C′**) Mean lifespan data of animals shown on panel (**C**). (**D**) Kaplan–Meyer lifespan curves of *eGFP-RNAi* (ON-target free RNAi control), versus *EDTP-RNAi*^*(V22)*^ and *EDTP-RNAi*^*(dsRNA)*^ (*EDTP* was downregulated only in dopaminergic neurons) animals. Flies were maintained at 29 °C. **(D′**) Mean lifespan data of animals shown on panel (**D**). In panels (**A)**, (**A′**), (**B′**), (**C′**) and (**D′**), the boxes represent the most typical 50% of the samples, the line indicates the median, upper and lower whiskers show remaining 25%-25% of the samples. Circles mark outliers. *P < 0.05, **P < 0.01, ***P < 0.001, statistical analysis was performed as described in the Materials and Methods, for statistics see Table S1.

Defective movement is a characteristic feature of aged flies^2,3,30,39,41^. Because locomotion is coordinated by neurons, we examined climbing ability in control versus EDTP defective (i.e., autophagy hyperactive) animals at different adult stages. Downregulation of *EDTP* was specifically achieved in dopaminergic neurons by using a *ple-Gal4* driver and two independent, effectively working RNAi constructs, *EDTP-RNAi*^*(V22)*^ and *EDTP-RNAi*^*(dsRNA)*^ (see the “Materials and methods” and Figs. S3A–D′, S4A–A′). Both treatments significantly increased the ability of animals to climb up on the wall of a glass vial within a certain period (Fig. 5A,A′, and Fig. S3A′). In case of the *EDTP-RNAi*^*(dsRNA)*^ construct, improvement in movement was evident even at later adult stages (day 21 and 28, Fig. 5A,A′). From these results we conclude that an age-associated decline in autophagic activity in specific neurons contributes to an impairment in locomotion of aged individuals, and this effect can be significantly delayed or attenuated by EDTP deficiency in the affected neurons. It is worth noting that in a control experiment, *EDTP* downregulation markedly increased, while *EDTP* hyperactivity lowered, the number of 2xFYVE-GFP-positive early autophagic structures in the affected cells at both young and old adult stages (Fig. S5).

In aged individuals, cytoplasmic proteins often become ubiquitinated, and this molecular mark assists the labelled factors to undergo proteasomal or autophagic degradation. We tested the age-related accumulation of ubiquitinated proteins in normal versus EDTP defective brain samples, by using a pan-neuronal *Appl-Gal4* driver that is active in essentially all neurons. In control samples, levels of ubiquitin-labelled structures significantly increased with age, and this change was effectively suppressed by *EDTP* downregulation (Fig. 5B,B′). Downregulation of *EDTP* increased the amount of mCherry-Atg8a- and GFP-Lamp1-positive structures (Fig. S3C–D′) and significantly decreased Ref(2)P levels in 21-day-old animals maintained at 29 °C, as compared with control (Fig. S4C–D′). Thus, enhancement of autophagic activity by inhibiting EDTP function protects neurons against the accumulation of damaged proteins, which is a general characteristic in various neurodegenerative pathologies.

Because autophagy plays a central role in the regulation of the ageing process^2–4,43^ and ageing is controlled by signalling systems that act in specific neurons^16^, we also tested the effect of dopaminergic neuron-specific *EDTP* downregulation on lifespan. *EDTP* was specifically downregulated during adulthood by using the *ple-Gal4* driver and two independent RNAi constructs (Fig. S3A–B′; animals were kept continuously at 25 or 29 °C). We observed that animals downregulated for *EDTP* live longer than control at both temperatures tested (Fig. 5C–D′ and Table S1). These results imply that enhancing autophagic activity in dopaminergic neurons by *EDTP* deficiency can lead to a longevity effect. In contrast, *EDTP* overexpression limits lifespan and interferes with climbing ability (Fig. S6A,A′).

### Accumulation of MTMR14 in brain neurons increases with age

**Figure 6:**
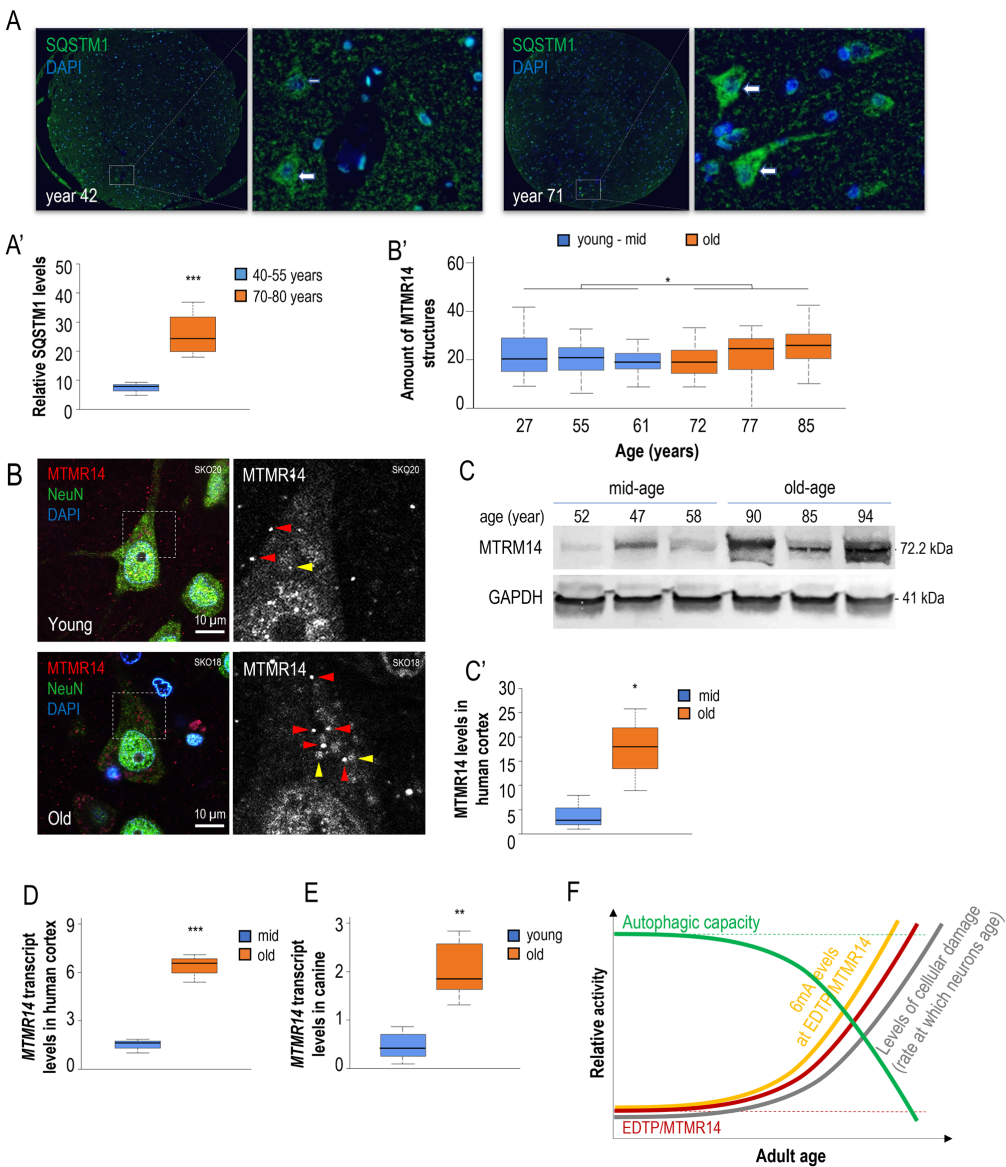
Human SQSTM1/p62 and MTMR14 accumulate with age in brain neurons. (**A)** Fluorescent images showing SQSTM1 accumulation (green) in post-mortem human brain samples at age of 42 (left) and 71 (right) years. White boxes indicate the enlarged area (at right). Images were captured with the same exposure time. DAPI staining (blue) indicates nuclei. A human SQSTM1-specific antibody was used for immunohistochemistry. (**A′**) Quantification of SQSTM1 levels in human brain samples at different adult stages. SQSTM1 accumulates more abundantly in aged samples relative to young ones. (**B**) NeuN (green)-MTMR14 (red) double-immunostained neurons with DAPI (blue) in the layer 3 of the temporal cortex (BA 38) of a „young” (up, SKO20, 27-year-old) and in an „old” (bottom, SKO18, 85-year-old) subject, photographed by a confocal fluorescence microscope. NeuN-immunopositive cells are green, MTMR14-immunopositive dots (small white arrows) are red, nuclei are blue. White dotted boxes indicate the enlarged area (at right), pictures of the right panel display the red channel (MTMR14 immunolabelling). Yellow arrowheads show autofluorescent lipofuscin (purple drops). Lipofuscin is present in both samples, but it is more abundant in the “old” subject. MTMR14-labelled dots (red arrowheads) are visible in cell bodies and dendrites. Scales bars correspond to 10 µm. (**B′**) Box plot of area covered by MTMR14-immunpositivity in percentage of cell area by cases. MTMR14-positivity was measured in the cells of the 3d layer of MTMR14-immunostained temporal cortical sections. The plot shows that the area of MTMR14-immunopositivity is higher in older subjects than in younger ones. Note the high individual variance among cells in most cases. The six subjects were divided into two groups as “young-middle ages” containing 27, 55 and 61 year-old subjects vs. “old” containing 72, 77 and 85 year-old subjects. The two groups are significantly different by *t*-test. (**C**) Western blot analysis showing that MTMR14 tends to accumulate with age in human cortex samples. GAPDH was used as an internal control. (**C′**) Quantification of EDTP protein levels in middle-aged and old groups, determined by the western blot analysis (**C**). *P < 0.05, **P < 0.01, ***P < 0.001. For statistics see Table S1. (**D**) RT-qPCR analysis demonstrates that *MTMR14* mRNA levels increase with age in human cortex. *GAPDH* was used as an internal control. Three middle-aged (47–58 years old) individuals and three old (85–94 years old) individuals were compared. Groups are significantly different by Mann–Whitney U-test, for more information of samples, see Table S5, ***P < 0.001. (**E**) RT-qPCR was performed to assess *MTMR14* mRNA levels in prefrontal cortex samples. 9 young (1–3 years) individuals and 6 old (13–17 years) individuals from various breeds were compared (for sample data, see Table S6). Commercial TaqMan assays were used to target the canine *MTMR14* orthologue (ThermoFisher, *Cf02682018_g1*). *GAPDH* (glyceraldehyde 3-phosphate dehydrogenase) was used as a reference gene (*Cf04419463_gH*). On the plot, the boxes represent the most typical 50% of the samples, lines indicate the median, upper and lower whiskers show remaining 25–25% of the samples. Circles mark outliers. **P < 0.01, independent two-sample *t*-test. (**F**) Model showing how EDTP/MTMR14 lipid phosphatases influence brain ageing. *EDTP/MTMR14* activity (red curve) gradually increases in neurons throughout the adult lifespan, thereby progressively downregulating autophagy as the organism age (green curve). As a consequence, cellular damage increasingly accumulates with age in neurons (grey curve). Green and grey dashed lines indicate relative physiological (basal) levels of autophagy and MTMR14/EDTP activity, respectively. Yellow line indicates relative 6 mA levels at *EDTP* locus. At later adult stages, the two lipid phosphatases act as endogenous pro-ageing factors.

To address the issue whether the regulatory role of this family of MTMR lipid phosphatases in brain ageing is evolutionarily conserved, we next monitored age-dependent changes in autophagic activity and MTMR14 accumulation in human brain neurons. p62/SQSTM1 levels were first determined in post-mortem human brain samples isolated at different (mid and old) adult ages. We found that the protein accumulates in brain neurons more abundantly in aged (70–80 years old) individuals than in younger (40–55 years old) ones (Fig. 6A,A′, and Table S2). Thus, a gradual decline in the capacity of autophagy may also occur during brain ageing in humans. In the light of this negative change in autophagic activity, one can explain why non-proliferating neurons tend to progressively accumulate cellular damage and become increasingly sensitive to demise over time, leading to the development of various neurodegenerative conditions at advanced ages.

In HeLa and C2C12 (mouse myoblast) cells, MTMR14 has been shown to localize to autophagic structures^22^. Here we tested the amount of MTMR14-positive particles in the brain cortex of differently aged human patients. Using a human MTMR14-specific antibody, an immunohistochemical analysis was performed, and results demonstrated that, similar to what we found in *Drosophila*, the amounts of MTMR14-labelled structures also elevate with age in brain neurons (Fig. 6B,B′, and Table S3). These results were confirmed by a parallel analysis applying another MTMR14-specific antibody on an independent set of brain samples derived from non-demented patients (Fig. S7A,A′, and Table S4). A subsequent western blot analysis was further conducted to quantify soluble MMR14 levels in human brain cortex samples, and we observed much higher intensities in aged samples (85–94 years old) relative to mid-age (47–58 old years) ones (Fig. 6C,C′, Fig. S7B, and Table S5). *MTMR14* transcript levels were also determined in these samples by using a qPCR analysis, and according to the results, the gene was expressed at higher levels in old samples than in young ones (Fig. 6D, Fig. S7C, and Table S5). So, the transcriptional regulation of *MTMR14* may contribute to enhanced levels (activity) of the gene product at advanced ages. These latter results correlate with human brain cortex-specific expression data freely available at GTExPORTAL (https://gtexportal.org), according to which MTMR14 is increasingly expressed with age in both sexes (Fig. S7D).

To reveal that *MTMR14* expression displays an age-related increase in the brain cortex of another mammalian species, we finally determined mRNA levels of *MTMR14* in young versus aged dogs (Fig. 6E and Table S6). Results showed that *MTMR14* is more active in aged animals relative to young ones. Taken together, lowered autophagic activity in neurons at advanced ages relative to young adulthood may be a consequence of increasing MTMR14 accumulation during lifespan.

## Discussion

In this study we showed that *Drosophila* Ref(2)P and human p62 proteins serving as a substrate for autophagic degradation progressively accumulate in the brain during ageing (Figs. 1B,B″″,C,C′, and 6A,A′). This gradual shift in Ref(2)P/p62 levels indicates an age-related decline in the capacity of autophagy in this organ, explaining why neurons increasingly accumulate cellular damage throughout the late adult stages and become sensitive to senescence and, subsequently, death. We further demonstrated that at advanced ages autophagy becomes impaired at two distinct stages. First, at an early stage when phagophore/autophagosome is formed (Fig. 1B–B″). Second, at a later stage when the autophagosome fuses with a lysosome or the autolysosomal content is degraded by acidic hydrolases (Fig. 1B–C″). These results suggest that impairment of autophagy during ageing is due to, at least in part, a regulatory (genetic) mechanism.

Autophagy plays a central role in ageing control^2–4,44^. It mediates the elimination of damaged cytoplasmic constituents, and its activity is influenced by various, if not all, longevity pathways, such as insulin/IGF1 (insulin-like growth factor) and TOR (kinase target of rapamycin) signalling, the mitochondrial respiratory system, and the molecular apparatus underlying caloric restriction^43^. Genes that downregulate autophagy in aged organisms certainly contribute to the deterioration of organs and tissues, thereby promoting the development of diverse age-associated degenerative diseases. However, the operation of these regulatory systems largely depends on environmental factors, such as food availability, oxygen concentration and temperature, and influences autophagy even in non-ageing cells, like germ line and cancer stem cells, in which autophagic degradation should not be fall off irrevocably. We propose that in ageing somatic cells, specific endogenous factors should set the rate at which the capacity of autophagy gradually declines during ageing, largely independently of environmental cues. Such a molecular clock factor that determines the rate at which cells age through modulating autophagic activity is Rubicon, which was shown recently to suppress progressively the process over the adult lifespan in divergent animal taxa^19^.

Why would autophagy become impaired in numerous neurons at late adult stages? In addition to random inactivating mutations in *Atg* genes, certain genetic factors may negatively regulate autophagy in aged adults. We demonstrated that *EDTP* gene, which codes for a conserved myotubularin-related lipid phosphatase interfering with autophagy by antagonizing PI3K/Vps34 (Fig. 2A)^23,25,34^, is also increasingly expressed in brain during the adult lifespan (Fig. 4A,B). EDTP protein also appeared to accumulate increasingly with age in this organ (Fig. 4C–C′). Its downregulation in neurons significantly triggered autophagy, improved locomotion and extended lifespan (Fig. 5, Figure S3C-D′ and Fig. S4A′). Consistent with these results, human MTMR14 was also found to accumulate at higher levels in human cortical neurons of aged patients compared with young ones (Fig. 6B–C′, Fig. S7A–B′). Furthermore, *MTMR14* expression increased with age (Fig. 6D, Fig. S7C,D). In dogs, *MTMR14* was similarly expressed at elevated levels in the brain prefrontal cortex of old adults relative to young ones (Fig. 6E). Together, similar to Rubicon, EDTP and MTMR14 progressively suppress autophagy during lifespan^19^. Both EDTP and MTMR14 perform this function at both early and late stages of the autophagic process (Fig. 1B–C″)^22^, while Rubicon does so at the latter exclusively^19^. Based on these data, one can conclude that the class III PI3K complex, which is regulated by EDTP/MTMR14 protein, may have been evolved as a primary molecular clock where autophagy can be suppressed in an age-dependent manner. Thus, the activity of the complex serves as a signature of ageing.

Ageing, a natural decline in the fitness and general physiology of an organism over time, is driven by the progressive accumulation of unrepaired cellular damage^1–4,44^. The process contributes to the elimination of post-reproductive adults from populations, thereby decreasing intraspecific competition under conditions of limited resources. Thus, the emergence of genetic factors promoting ageing can strengthen the long-term subsistence of species at the expense of individual lives. As part of organismal ageing, the gradually increasing disintegration of neuronal functions progressively limits the ability of individuals to survive. In this study, we uncovered a novel regulatory mechanism, by which the brain deteriorates at an increasingly growing rate during the adult lifespan. We demonstrated that *Drosophila* EDTP and human MTMR14, two conserved negative regulators of the autophagic process^22,23,34^, progressively accumulate in brain neurons throughout adulthood (Figs. 4, 6B–C′). Hence, these orthologous proteins, after passing through a critical accumulation level in neurons at a certain adult stage, function as endogenous pro-ageing factors that promote brain ageing and restrict lifespan by increasingly downregulating autophagy over time (Fig. 6F). Owing to the waning autophagic capacity, neurons become progressively sensitive to accumulate cellular damage and, as a consequence, to death during the adult lifespan.

The conserved MTMR lipid phosphatases EDTP and MTMR14 act as endogenous factors that increasingly lower the autophagic activity during lifespan, thereby representing a novel class of endogenous pro-ageing regulatory factors. The function of EDTP and MTMR14 in ageing control appears to be similar to that of Rubicon^19^. In sum, data presented in this study reveal a novel mechanism that drives brain ageing. We suggest that the gradually growing sensitivity of neurons to demise during ageing is genetically determined. Brain ageing, at least in part, is a regulated process, and to acquire a neurodegenerative condition is simply a question of time the individual lives for.

Previously, we found that both EDTP and Mtmr6, the fly orthologues of mammalian MTMR14 and MTMR6-8, respectively, function in autophagy control^21,45,46^. These proteins exert their regulatory role in a condition-dependent manner in the larval (L3F stage) fat body; EDTP inhibits basal autophagy but does not influence stress-induced autophagy while Mtmr6 promotes basal autophagy but inhibits the process under various stress conditions. In mammals, MTMR14 and MTMR8 each convert PI3P to PI, while MTMR6 blocks PI(3,5)P2 generation^38^. Under both starving and nutrient rich conditions, MTMR14 inhibits autophagy whereas MTMR6 influences starvation-induced autophagy only ^22^. Thus, MTMR14 and MTMR6 have distinct roles in different model systems. In the future, it is worth studying the functional relationship of these proteins in the adult nervous system and in lifespan determination.

## Materials and methods

### Fly stocks, genetics and conditions

Flies were kept at 25 °C or 29 °C on normal fly cornmeal nutrient. Strains were ordered from Bloomington Drosophila Stock Center (BDSC) and Drosophila Genetic Resource Center, Kyoto (DGRC), or were kindly provided by another researcher or generated and described by us earlier.

For immunohistochemistry and fluorescence microscopy, *w[1118]* (BDSC: 5905) and *UAS-GFP-2xFYVE (II)* (BDSC: 42712) animals (crossed with *Appl-Gal4*) were used, respectively. Endogenous *GFP-Atg8a* expression was described in Ref.^31^.

For western blot analysis, *w[1118]* was used as control. For *EDTP* overexpression, *EDTP[GSV6]* (DGRC: 202239) was used, under the control of *Appl-Gal4* (BDSC: 32040). *EDTP[MI008496]* (BDSC: 44782) was used as a hypomorphic allele.

Animals of *w[1118]; 3xmCherry-Atg8 és w[1118];* + *; UAS-GFP-Lamp1* genotypes were provided by Gábor Juhász (Eötvös Loránd University Budapest, Hungary). For lifespan assays, y[1]* v*[1]*, Appl-Gal4; EDTP TRiP /tubGal80[ts]* and *y*[1] sc[*]v[1]*, Appl-Gal4; eGFP TRiP /tubGal80[ts]* (control) animals were created by crossing *Appl-Gal4*, *tubGal80[ts];TM2/TM6B* (BDSC: 7108), *eGFPTRIP (V22)* (BDSC: 41550) and *EDTP TRIP (V22)* (BDSC: 41633) animals. In case of another lifespan assay, and for climbing assays, animals with *w[1118]/*+*;* +*; pleGal4/*+ and w[*]/+*;* +*; UAS-EDTP-RNAi/pleGal4* genotypes were created by crossing *pleGal4* (BDSC: 8848), *w[1118], eGFPTRiP (V22)*, *EDTP TRiP (V22)* and w[*]; UAS-EDTP-RNAi (III) animals, which were a gift from Tamás Lukácsovich (Department of Developmental and Cell Biology, University of California, Irvine, CA, USA) and described in Manzéger et al.^47^. Flies were kept at 29 °C and dead animals were counted daily.

For lifespan measurements, climbing assays and protein ubiquitination tests, two different RNAi construct were used to downregulate *EDTP* expression. BDSC: 41633 (EDTP-RNAi^(V22)^) strain contains a shorter target sequence: CAG TAG TGT AAT AGT AAT CAA (Fig. S2B), while the other construct (EDTP-RNA^dsRNA^) contains a longer but different target sequence (Fig. S2B): ctc gag GGT ACC GGG AAA TGG ACT CTT CGG GCA AGT TGG GGG AGT GGG AGG TGG AGG CTC CTC GGG AAC AAC CGC CAC TGC CAC GCC TCT GAA CAG CAG TGC AGG AAG CAC CGG AAG TGA GGG TGT GGG CAT CCA AGC CTT TGT GAC CTT TGC CAA TCC CCT GCA GAC GCA ACA ACA GCA TCC GCT CCA GCA ACA ATA TCC CTC GCA GCA GAT GCA TCC CCT CCA CGC GCA ATA TCC CTC CCA GCA GCC ACA TCC ACT CCA GCA GCA GCA GCA GCA GCC ATC GCA ACA GCA ACC ACA AAA TAC GAT ATA CGA GGA TCA GTA TGA TAT CCA GCG AAT GCG GGA ATT GGT AAC GAT GGC CAA ATA TGC GAG ATG CCG TCA AAG ATT CGC CGT GCC TGT GAT TAT GTA TCG CGG AAA GTA CAT ATG CCG CTC TGC CAC GCT ATC CGT CAT GCC AGA AAC CTA CGG CCG AAA AGT GGT GGA CTA TGC CTA CGA CTG CCT GAG TGG CGG CAA TTA CAC CGC GCC AAA CGG AGA AGA GAA CGA TGC TGA CTC CAC GGA CGA GTC GCT GAT CAC CCA CAT GCA CGA CCA GGC GCA GTC GCA GTT CAG CTA CGA CGA AGT CAT CAA GAG TGA CAT CCA GCT GCT GCA TAC GCT CAA TGT CTC AAC CAT TGT GGA CCT CAT GGT CGA AAA CCG CAA AAT CAA ATA CTT CAT GGC aga tct.

For studying *EDTP* expression, y[1] w[*]; Mi{Trojan-GAL4.0}EDTP[MI08496-TG4.0]/ P{y[+ *t7.7] w[*+ *mC]* = *10XUAS-IVS-myr::GFP}su(Hw)attP5* and y[1] w[*]; Mi{Trojan-GAL4.0}EDTP[MI08496-TG4.0]/ P{w[+ *mC]* = *UAS-GFP.nls}14* animals were created by crossing *EDTP TrojanGal4* (BDSC: 66899), *UAS-myrGFP* (BDSC: 32199) and *UAS-GFPnls* (BDSC: 4775) strains. For measuring mCherry-Atg8a-labelled autophagic structures, *UAS-mCherry-Atg8a* transgene was applied, kindly provided by Gábor Juhász (Department of Anatomy, Cell- and Developmental Biology; Eötvös Loránd University, Budapest, Hungary) and described in Ref.^48^.

### Immunohistochemistry and fluorescence microscopy on Drosophila samples

To determine Atg8a levels, a GFP-Atg8a (p-Atg8a-eGFP-Atg8a) reporter construct was used^31^. Samples were prefixed with 4% formaldehyde (solved in PBS) and washed three times (for 10 min) in PBS. Nuclei were stained with 50 µg Hoechst in glycerol:PBS (4:1) cover solution. During measurement, we used the same exposition time and magnification for all samples.

Fixation and immunohistochemistry were performed according to Ref.^46^. The following antibodies were used: anti-Ref(2)P 1:200, rabbit—a gift from Gábor Juhász, Department of Anatomy, Cell- and Developmental Biology, Eötvös Loránd University, Budapest, Hungary^49^ and anti-Atg5 (1:500, rabbit, Sigma Aldrich, AV54267), anti-Ubiquitin (1:500, mouse, Merck, ST1200). The following secondary antibodies were used: anti-Rabbit Alexa Fluor 488 (1:500, Life Technologies, A11008), anti-Mouse Alexa Fluor 488 (1:500, Life Technologies, A11001). Nuclei were stained by Hoechst dye (0.1 mg/ml, Molecular Probes, 33342).

### Fluorescent microscopy on human samples

Fluorescent images were captured with a Zeiss Axioimager Z1 upright microscope (with objectives Plan-NeoFluar 10 × 0.3 NA, Plan-NeoFluar 40 × 0.75 NA and Plan-Apochromat 63 × 1.4 NA) equipped with ApoTome, and a Nikon C2 confocal microscope (with objective 60 × Oil Plan APO VC NA = 1.45). AxioVision 4.82 and Jmage J 1.52c software were used to examine and evaluate data obtained. We calculated Pearson's coefficients by Image J 1.52c, for evaluating the colocalization of mCherry-Atg8a and GFP-Lamp1 particles.

### Western blotting

Western blot samples were prepared from 10 female heads, which were treated in 32 μl of Fly Lysis buffer + 32 μl 2 × Laemmli buffer. 15 µl samples were run on 4–20% Mini-PROTEAN^®^ TGX™ Gel and blotted onto Nitrocellulose Membrane (Kisker Biotech, 40520100). After blocking with 3% Milk Powder (BioRad 170-6404 /Blotting-Grade Blocker/) dissolved in TBST, membranes were probed with specific antibodies [anti-Tubulin (1:1000, mouse, Sigma T6199), anti-Ref(2)P (1:2000, rabbit^48^), anti-Atg8a (1:2500, rabbit^50^), anti-EDTP, 1:1000, rat^22^, anti-mouse IgG alkaline phosphatase (1:1000, Sigma, A8438), and anti-rabbit IgG alkaline phosphatase (1:1000, Sigma, A3687), anti-rat IgG alkaline phosphatase (1:1000, Sigma, A5153), and developed by NBT-BCIP solution (Sigma, 72091). Each western blot analysis was repeated at least three times with independent biological samples.

### Quantification of EDTP transcript levels

Isolation of total mRNA from heads of adult flies at age of 1, 10, 20, 30, 40, 50 and 60 days was performed according to the Direct-zol™ RNA MiniPrep kit (Zymo Research, R2050) protocol, then cDNA was generated by RevertAid RT Reverse Transcription Kit (Thermo Scientific, K1691). Quantitative Real-Time PCR reactions were performed in a Roche LightCycler 96 Instrument (Roche Molecular Systems) with FastStat Essential DNS Green Master kit (Roche, 06924204011). Quantitative measurements were repeated three times using newly isolated samples, and each qPCR experiment contained three technical repeats. GAPDH mRNA level was used as an inner control. Forward (F) and reverse (R) primers were as follows: *EDTP* F: 5′-AAA AAG CTC CGG GAA AAG G-3′ and R: 5′-AAT TCC GAT CTT CGA CAT GGC-3′, *GAPDH* F: 5′-TAC TTC ATG GCC GTT TCC TC-3′ and R: 5′-AGA TCC CAA TCC CGG TAC TC-3′.

### Determination of relative N^6^-methyladenine levels

Genomic DNA was isolated from *Drosophila* at different adult stages according to standard protocols (Thermo Scientific GeneJET Genomic DNA Purification Kits #K0721 and #K0722). Samples were digested with DpnI at 37 °C for 20 min, then the enzyme was inactivated at 80 °C for 20 min, then a PCR experiment was performed as described previously by Yao et al.^42^. Forward and reverse primers, and PCR conditions were as follows. For *Drosophila*, control: 5′-TGA GGA ACA TCA TTC TTG GCT C-3′ and 5′-CTA CGG GGA GCT GAT GTA CT-3′; *6mA EDTP*: 5′-ACC GTT AGG TCA GAT CTA TCC AG-3′ and 5′-CTA CGG GGA GCT GAT GTA CT-3′. PCR: 95 °C for 30 s, then 95 °C for 10 s and 58.8 °C for 30 s repeated by 30/50 (control/sample) cycles.

### Climbing assays

20 adult flies (which were raised at 29 °C) expressing the transgene under the control of *pleGal4* driver were anesthetized, and placed into a vertical glass column (length, 25 cm; diameter, 1.5 cm). After 1 h of recovery period from CO_2_ exposure, flies were gently hit 5 times to the bottom of the column. The number of flies that reached the line at 21.8 cm height within 20 s was counted. Three series of two parallel measurements were performed in each experiment. Scores represent the mean number of flies that reached the top against the total number tested. Results are presented as mean ± S.D.

### Lifespan assays

For lifespan measurements, an equivalent number of males and females was used. Animals were transferred into fresh nutrient-containing vials at every second day. The number of dead animals was counted daily. Measurements were carried out with five parallels. Tests were carried out at 25 and 29 °C.

### Statistical analysis

For statistical analysis of climbing assays, lifespan measurements (mean lifespan) and fluorescence microscopy, results were determined by using R Studio (Version 3.4.3). The distribution of samples (normal or not) was tested with Lilliefors-test. If it was normal, *F*-test was performed to compare variances. In cases when variances were equal, two-samples *t*-test was used, otherwise *t*-test for unequal variances was applied. In case of non-normal distribution, Mann–Whitney U-test was performed. For lifespan curve statistics, the logrank (Mantel-Cox) method was used, calculated with the SPSS17.0 program.

### DAB immunohistochemistry and image analysis on human post-mortem brain samples

Ubiquitinated proteins in the autophagic-endocytotic pathway and autophagy impairment were observed by immunohistochemical localization of anti-myotubularin-related phosphatase MTMR14 antibody, respectively. After deparaffinisation and rehydration, sections were boiled in 0.01 M citrate-buffer solution (pH 6.0) in a microwave oven for 2 min (set at 900 watts) for antigen recovery. After blocking the endogenous peroxidase in 0.1 M TBS containing 3% H_2_O_2_ for 10 min at 37 °C, sections were washed for 3–5 min in 0.1 M TBS (pH 7.4) at RT. Tissue sections were next permeabilised, and the background binding of antibodies was reduced in a blocking solution (0.1 M TBS containing 5% normal goat serum, 1% BSA, 0.05% Triton X-100) for 30 min at 37 °C. Sections were covered with the above solution containing either mouse anti-NeuN primary antibody (1:500 final dilution; Chemicon, Billerica, MA, USA), rabbit polyclonal anti-MTMR14 primary antibody (1:100 final dilution; ab102575, Abcam, Cambridge, UK) overnight at 4 °C. After incubation with the primary antibodies, sections were washed for 4 × 5 min in 0.1 M TBS (pH 7.4) at RT. Negative control experiments were performed when the appropriate primary antibody was omitted. Sections were then treated with either biotinylated anti-rabbit or anti-mouse IgG secondary antibody (1:200 final dilution; Amersham Biosciences, Little Chalfont, Buckinghamshire, England) in a blocking solution (where Triton X-100 was omitted) for 5 h at RT. After several washes (4 × 5 min), biotinylated streptavidin-peroxidase tertiary antibody (1:200 final dilution; Amersham) in a blocking solution (without Triton X-100) was applied to the sections overnight at 4 °C. Sections were washed again in 0.1 M TBS (pH 7.4) for 4 × 5 min at RT, and processed for peroxidase enzyme histochemistry using Sigma Fast DAB Tablet (Sigma, St. Louis, MO, USA) according to the manufacturer’s protocol. Sections were washed for 3 × 5 min in 0.1 M TBS (pH 7.4) at RT, rinsed in distilled water for 1 min, dehydrated in a series of ethanol solutions, covered with DPX mounting medium (Fluka, 30 Buchs, Switzerland) and coverslipped.

Digital images from sections of temporal cortices of 4 non-demented subjects (see Table S4 for ages, sexes, post-mortem delays and Braak stages) immunostained for NeuN were taken with a Leica DMLB light microscope (Leica Microskopie und Systeme GmbH; Wetzlar, Germany) using a Qimage MicroPublisher 3.3 RTV digital camera (Surrey, BC, Canada). NeuN-positive cells (not shown) were counted with the use of the computer program ImageJ (version 1.47; developed by W. Rasband at the U.S. National Institutes of Health, and available from internet at http://rsb.info.nih.gov/ij) as we published earlier (for details, see Refs.^35,51^. MTMR14 immunoreactivities were quantified in lipofuscin-free cytoplasm through use of ImageJ image processing software. A total of 134 cells from non-demented samples were analysed. Measurements were taken by two independent investigators and the density values were averaged.

### p62/SQSTM1 fluorescent antibody staining on human post-mortem brain samples

Protein distribution was measured using immunofluorescent techniques, using fluorophore‐ tyramide signal amplification method (Perkin Elmer, Waltham, MA, USA). A BOND‐RX automated stainer (Leica Biosystems, Wetzlar, Germany) was used to prepare the slides for staining. Sections were “baked” (30 min at 60 °C), dewaxed using Bond Dewax Solution (Leica Biosystems, 72 °C), and run through a heat‐induced epitope retrieval step (EDTA‐based solution, pH 9.0, 20 min at 100 °C).

Following this “pretreatment,” slides were manually washed in phosphate‐buffered saline (PBS), incubated in 0.03% H_2_O_2_ for 30 min to block endogenous peroxidase, and washed again. Primary rabbit antibody raised against human SQSTM1 (HPA003196, Atlas Antibodies, Stockholm, Sweden) diluted 1:10 in primary antibody buffer (0.3% TX‐100, 0.1% NaN_3_, PBS) and added to the slides for overnight incubation in a humidified chamber at 4 °C. The following day, slides were washed in Tris‐buffered saline (TBS, pH 7.4)–Tween 20, and blocked in Tris‐NaCl blocking buffer (TNB) (0.1 mol/L Tris‐HCl, pH 7.5, 0.15 mol/L NaCl, 0.5% blocking reagent, Perkin Elmer) for 30 min. The secondary swine anti rabbit HRP conjugate antibody (DAKO) diluted 1:200 in TNB were then applied to slides for 30 min, followed by a wash in TBS‐Tween 20. For the tyramide signal, amplification slides were incubated with fluorescein‐conjugated tyramide diluted (1:100) in amplification reagent (Perkin Elmer) for 15 min at room temperature.

To quench lipofuscin autofluorescence in the tissue, slides were counterstained with lipophilic Sudan Black B solution (1% w/v in 70% ethanol, Sigma‐Aldrich, St. Louis, MO, USA) for 5 min. The slides were then dipped in 70% ethanol followed by a PBS wash and coverslipped using an aqueous mounting medium containing a 4′,6‐diamidino‐2‐phenylindole (DAPI) counterstain (ProLong Gold Antifade Mountant with DAPI, ThermoFisher Scientific, Waltham, MA, USA). Unless otherwise noted, all steps were executed at room temperature.

Images were acquired on an automated VSlide slide scanning system (Metasystems, Altlussheim, Germany). Entire pieces of tissue on the slides were imaged with a 20 × objective (NA = 0.45, resolution 3.6 pixels/μm). Each field of view was captured at 3 z‐levels with a 1 μm interval to create an extended focus image. Acquired field of view images were stitched to create a complete overview with microscopic resolution. The emission spectra for the fluorophore‐conjugated secondary antibodies were as follows: Hoechst (420–485 nm), Cy2 (490–530 nm), Cy3 (550–570 nm), Cy3.5 (580–595 nm), and Cy5 (650–670 nm).

### Quantification of MTMR14 transcript levels in human cortical samples

Total human mRNA samples were isolated from the temporal cortical tissues according to the Direct-zol™ RNA MiniPrep kit (Zymo Research, R2050) protocol, then cDNA was generated by RevertAid RT Reverse Transcription Kit (Thermo Scientific, K1691). Roche LightCycler 96 Instrument (Roche Molecular Systems) with FastStat Essential DNS Green Master kit (Roche, 06924204011) was used for quantitative Real-Time PCR reactions. *GAPDH* was used as an internal control. The following forward and reverse primers were used: GAPDH: 5′-TCG GAG TCA ACG ATT TGG T-3′ and 5′-TTC CCG TTC TCA GCC TTG AC-3′, MTMR14: 5′-GTA ACG GGC TGT GGC AGT AT-3′ and 5′-TTC CCG TTC TCA GCC TTG AC-3′.

### Measurement of human cortical MTMR14 protein levels

Proteins were isolated from temporal cortical tissues according to the standard sample preparation protocol for Western blot of Abcam. 15 mg tissue was homogenized per each sample in 600 µl lysis buffer (included RIPA buffer and protease and phosphatase inhibitors) with electric homogenizer. 20 µl samples were run on 4–20% Mini-PROTEAN^®^ TGX™ Gel and blotted onto Nitrocellulose Membrane (Kisker Biotech, 40520100). After blocking with 3% Milk Powder (BioRad 170-6404 /Blotting-Grade Blocker/) dissolved in TBST, membranes were probed with specific antibodies [anti-GAPDH (1:2000, rabbit, Sigma G9545), anti-MTMR14 (1:500, rabbit, Abcam ab102575), anti-rabbit IgG alkaline phosphatase (1:1000, Sigma, A3687), and developed by NBT-BCIP solution (Sigma, 72091).

### MTMR14 fluorescent staining on human cortical tissues and quantification of reactions

Control human temporal cortical tissue was obtained from five males and one female (SKO20: 27-year-old, SKO7: 55-year-old, SKO19: 61-year-old, SKO11: 77-year-old, SKO16: 72-year-old, SKO18: 85-year-old) subjects died from causes not related to any brain disease, and had no history of any neurological disorder. Control subjects were processed for autopsy at the Department of Pathology, Saint Borbála Hospital, Tatabánya, Hungary. Tissues were obtained and used in a manner compliant with the Declaration of Helsinki. All procedures were approved by the Regional and Institutional Committee of Science and Research Ethics of Scientific Council of Health, in accordance with the Hungarian Law (ETT TUKEB 15032/2019/EKU).

Brain samples were removed 2–4, 5 h after death, both internal carotid and vertebral arteries were cannulated, and were perfused first with physiological saline (1.5 l in 30 min) containing 5 ml of heparin, followed by a fixative solution containing 4% paraformaldehyde, 0.05% glutaraldehyde and 0.2% picric acid in 0.1 M PB, pH 7.4 (4–5 l in 1.5–2 h). Temporal cortex was removed after perfusion, and post-fixed in the same fixative solution overnight, but without glutaraldehyde^52^. Subsequently, 60 µm-thick coronal sections were prepared from the blocks with a Leica VTS-1000 Vibratome (Leica Microsystems, Wetzlar, Germany) for immunohistochemistry. Sections were washed in PB, and immersed in 30% sucrose for 1–2 days, then freeze-thawed three times over liquid nitrogen. Sections were processed for immunostaining as follows: after thoroughly washed in PB for five times, endogenous peroxidase activity was blocked by 1% H_2_O_2_ in TRIS buffered saline (TBS, pH 7.4) for 10 min. TBS was used for all washes (3 × 10 min between each antiserum) and for dilution of the antisera. For confocal microscopic investigations, the incubation of primary antibodies happened simultaneously (anti-MTMR14, rabbit, 1:500; ABCAM, #ab102575), anti-NeuN, mouse, 1:2000; Merck, #Ab377). After incubation, secondary antibodies with fluorophores were applied (DAM Alexa488 1:500; Thermofisher, #A-2107, DAR Alexa594 1:500; Thermofisher, #A-2102) for 3 h, then samples were incubated with DAPI fluorophore (1:10,000, Sigma-Aldrich, #D9564) for 2 h. For reducing autofluorescence, samples were incubated with CuSO4 solution for 40 min or samples were treated with AER (Autofluorescence Eliminator Reagent; Merck, #2160) in 70% ethanol for 5 min. After that, samples were mounted in Aqua-Poly/Mount (Polysciences, #18606-20). To avoid any interaction with additional chemicals causing reduced fluorescent intensity, quantitative measurement was carried out without AER. Samples were analysed by using a Nikon C2 confocal fluorescent microscope with 60 × oil objectives. ROI (region of interest) areas were defined from total width of the cortical layer 3 from temporal cortical (Brodmann’s area 38) sections. NeunN-labelled cells were photographed in their largest perikaryal extent. With this method, ca. 30 cells were photographed from the 3d-layer of each cortical samples. NeuNm/MTMR14r/DAPI triple fluorescent confocal images were analysed by ImageJ 1.50b program. During measurements, the three channels (green: 488 nm; red: 594 nm; blue: DAPI 470 nm) were visualized separately. Cytoplasm areas without nucleus or autofluorescent lipofuscin were determined using the NeuN (green) and DAPI (blue) channels. In these cells, one or two 2–4 µm^2^ ROI areas were designated. Intensity was measured in ROI areas in the red channel corresponding to MTMR14 immunolabelling. Although the imaging parameters were uniform, absolute intensity values were not comparable due to individual differences in the samples, so a relative intensity unit (IU) was determined for each individual cell. As a reference intensity, the relative intensity (measured as described above) of glial cells adjacent to neurons was used. The relative intensity was calculated based on the following formula: IU = (AI-RI)/(MI-RI) × 100, where AI is the absolute intensity measured in the cytoplasm of neurons, RI is the reference intensity, MI is the highest intensity found in the image. For images without identifiable glial cells, the average of the RI values of the images of the subject was used for the calculation. For statistics, the diagrams Statistica 13.4 program Microsoft Excell was used. The six subjects were divided into two groups, as “young” containing the 27, 55 and 61 year-old subjects vs. “old” containing the 72, 77 and 85 year-old subjects. Normality of obtained IU values was checked by Kolmogorov–Smirnov test. Based on *P* > 0.2 values, data were normally distributed, so pooled intensity unit data of the two groups were compared by T-test.

### Measuring MTMR14 transcript levels in the prefrontal cortex of dogs

Total RNA was isolated from canine frontal cortical samples stored frozen in RNAlater (Thermo Fisher Scientific, #AM7021), by using TRIzol (ThermoFisher Scientific, #15596018), according to the manufacturer instructions. Prior to immersing tissue pieces in TRIzol, each piece was rinsed in 1 ml of sterile PBS in a new tube, and centrifuged for 5 min at 500*g*. TRIzol was added to samples after removing PBS. Tissue pieces were homogenized in TRIzol by an Ultra-Turrax homogenizer (Ika). Following homogenization, RNA isolation took place. The quality of isolates was checked by agarose gel electrophoresis, and concentrations were measured by a NanoDrop device (ThermoFisher Scientific). Isolated RNA samples were stored at − 20 °C prior to cDNA synthesis, and at − 80 °C for long term storage.

1000 ng of total RNA was reverse-transcribed into cDNA, using Maxima RevertAid cDNA Synthesis Kit (Thermo Fisher Scientific, #K1672). Reverse transcription was performed, using random hexamer primers. Then, cDNA samples were diluted tenfold in nuclease-free water, and kept either at − 20 °C or at − 80 °C. Quantitative Real-Time PCR reactions were performed in a Roche LightCycler 96 Instrument (Roche Molecular Systems), using commercial TaqMan assays and TaqMan Gene Expression Master Mix (Thermo Fisher Scientific, #4369514). The canine orthologues of *MTMR14* and *GAPDH* (internal control) are *Cf02682018_g1* and *Cf04419463_gH*, respectively. Reactions were run in triplicates in 96-well plates.

### Ethics declarations and compliance of the ARRIVE guidelines

Procedures involving experimentation on vertebrate animal subjects were done in accord with the guide of Eötvös Loránd University, Budapest, Hungary. We confirm that the studies presented in the manuscript were carried out in compliance with the ARRIVE guidelines.

### Legal approval for human experiments for using the data of the subjects

Animal and human brain samples handling was performed according to the guidelines of the Committee on human Experimentation of University of Szeged, Faculty of Medicine and Faculty of Science and Informatics (Szeged, Hungary), as well as Institute of Experimental Medicine, Hungarian Academy of Sciences (Budapest, Hungary), in which the experiments were performed. We confirm that an informed consent was obtained from all subjects and/or their legal guardian (for using them data in our manuscript).

## Supplementary Information

Supplementary Information.

## Data Availability

Animal and human brain samples handling was performed according to the guidelines of the Committee on human Experimentation of University of Szeged, Faculty of Medicine and Faculty of Science and Informatics (Szeged, Hungary), as well as Institute of Experimental Medicine, Hungarian Academy of Sciences (Budapest, Hungary), in which the experiments were performed. The datasets used and/or analysed during the current study available from the corresponding author on reasonable request.

## Abbreviations

Atg: Autophagy-related
Beclin 1: Coiled-coil, myosin-like BCL2-interacting protein
EDTP: Egg-derived phosphatase
FYVE zinc finger domain: Fab 1 (yeast orthologue of PIKfyve) YOTB, Vac 1 (vesicle transport protein) and EEA1
GFP: Green fluorescent protein
IGF: Insulin-like growth factor
AD, LC3B: Microtubule-associated proteins 1A/1B light chain 3B
MTM: Myotubularin
MTMR: Myotubularin-related
PI: Phosphatidylinositol
PI3K: Class III phosphatidylinositol 3-kinase
PI3P: Phosphatidylinositol 3-phosphate
PI3,5P_2_: Phosphatidylinositol 3,5-bisphosphate
Ref(2)P: Rubicon (RUN domain and cysteine-rich domain containing, Beclin 1-interacting protein)
SQSTM1: Sequestosome 1
TOR: Target of rapamycin
UVRAG: Ultraviolet irradiation resistance-associated gene
Vps34: Vacuolar protein sorting 34
6mA: N6-methyladenine
