# Supplementary material for: A conserved MTMR lipid phosphatase increasingly suppresses autophagy in brain neurons during aging
Source: Sci Rep. 2022 Dec 17;12:21817. doi: 10.1038/s41598-022-24843-w (PMC9759524; doi:10.1038/s41598-022-24843-w)
Supplement: Supplementary file 1 — Supplementary Information. [file 41598_2022_24843_MOESM1_ESM.docx]

**Supplementary Materials**

**A conserved MTMR lipid phosphatase increasingly suppresses autophagy in brain neurons during aging**

**Tibor Kovács, Janka Szinyákovics, Viktor Billes, Gábor Murányi, Virginia B. Varga, Annamária Bjelik, Ádám Légrádi, Melinda Szabó, Sára Sándor, Enikő Kubinyi, Cecília Szekeres-Paracky, Péter Szocsics, János Lőke, Jun Mulder, Balázs Gulyás, Éva Renner, Miklós Palkovits, Károly Gulya, Zsófia Maglóczky and Tibor Vellai**

**Supplementary Figures S1 to S8**

**Supplementary Tables S1 to S6**

**Figure S1.**

**
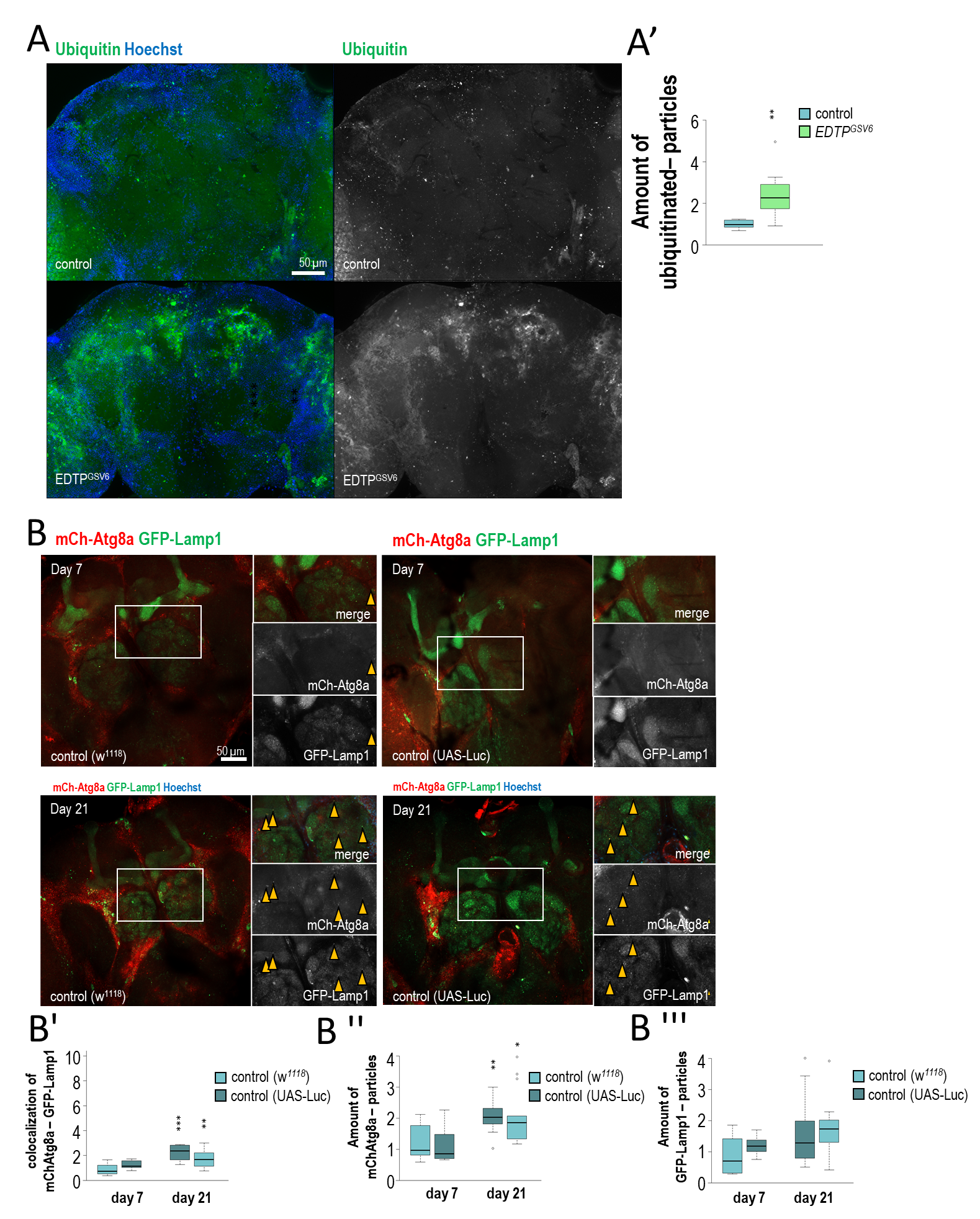
**

**Figure S1. The amount of autolysosomes increases with age in brain neurons.** (**A-A’**) Relative amount of ubiquitinated structures in EDTP-overexpressing (*EDTP^GSV6^*), 21-day-old animals maintained at 29°C. The amount of ubiquitin-positive structures (green) elevates in response to EDTP hyperactivity. Hoechst staining (blue) indicates nuclei. (**B-B’’’**) Co-localisation of mCherry-Atg8a (autophagic structures) and GFP-Lamp1 (lysosomal compartments) reporters in animals at the stage of 7 and 21 days. More co-labelled structures (yellow arrows) are visible in old animals (**B’**). On the plot, the boxes represent the most typical 50% of the samples, the line indicates the median, upper and lower whiskers show remaining 25%-25% of the samples. Circles mark outliers. *: P<0.05; **: P<0.01; ***: P<0.001 at each comparison with day 1. For statistics, see the Materials and Methods.

**Figure S2.**


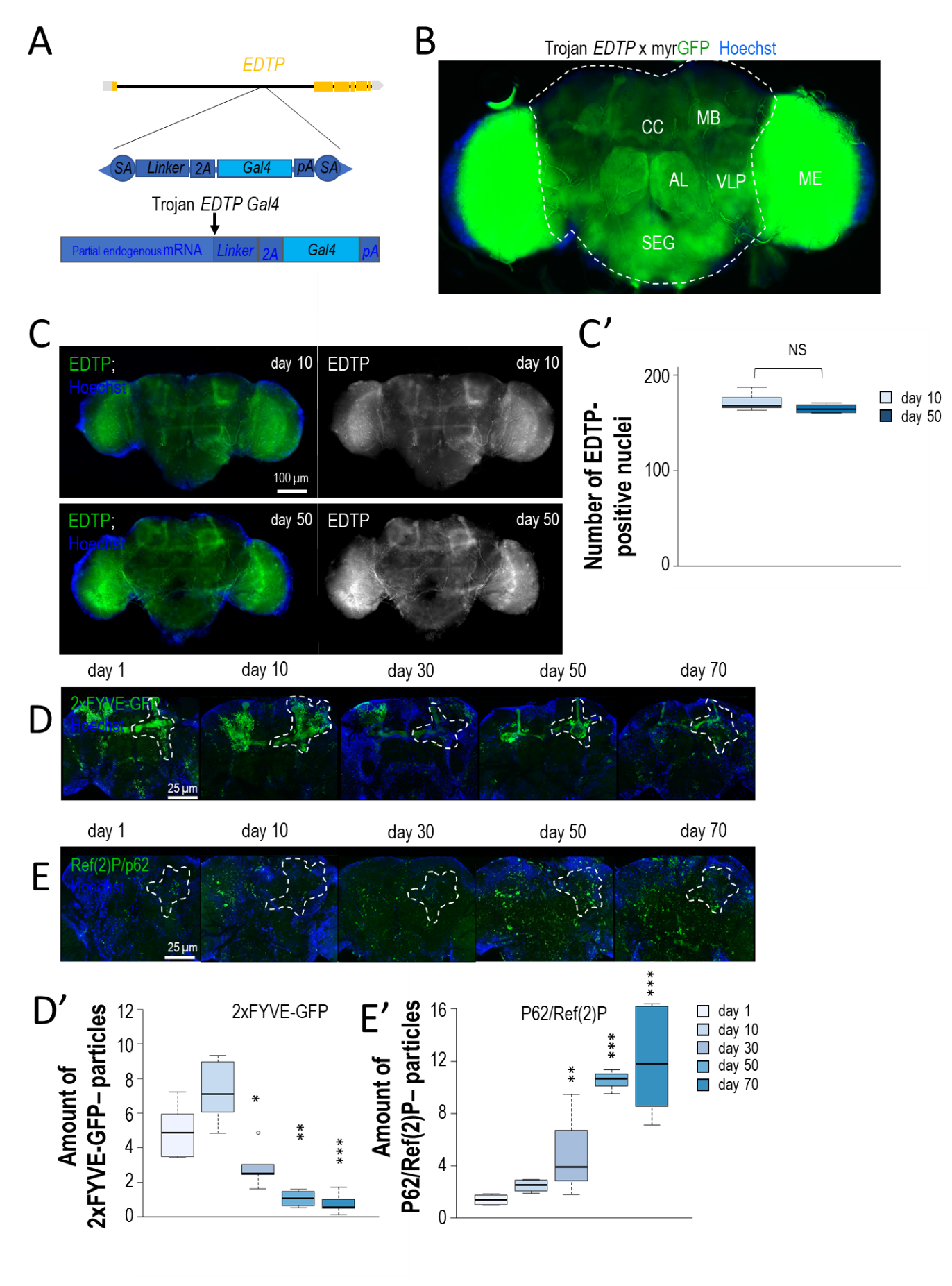


**Figure S2. Expression of a Trojan *EDTP*-*GFP* gene trap reporter system in the *Drosophila* brain**.  **(A)** Structure of the Trojan-*EDTP*-*Gal4* gene trap system used in this study. A *Gal4* driver, which activates a *UAS-GFP* reporter, was inserted into the first intron of *EDTP* gene, and serves as an artificial exon containing a STOP codon (*i.e*., *GFP* transcription is controlled by the endogenous *EDTP* regulatory sequences; *UAS-myr-eGFP* was expressed). **(B)** Expression of a Trojan-*EDTP*-*Gal4* driver in the brain. Dashed line indicates the midbrain, where relative EDTP levels were measured. AL, antennal lobe; MB, mushroom body; CC, central complex; ME, medulla; VLP, ventrolateral protocerebrum; SEG, subesophageal ganglion. Medullas were excluded from the analysis. **(C)** The amount of cells expressing *EDTP* does not change with age. UAS-GFP.nls (nuclear localization signal-tagged GFP) was driven by Trojan-*EDTP-Gal4*. Left: fluorescent images, right: the corresponding uncoloured versions. **(C’)** Quantification of cells expressing *EDTP* in the brain at different stages of adulthood (10 vs. 50 days). *EDTP* expression is increased during ageing in the area of mushroom body (MB). (**D-D’**) The amount of 2xFYVE-GFP-positive structures is lowered in old samples relative to young ones. (**E-E’**) Ref(2)P levels increase in the MB in old animals compared with young ones. On the plot, the boxes represent the most typical 50% of the samples, the line indicates the median, upper and lower whiskers show remaining 25%-25% of the samples. Circles mark outliers. *: P<0.05; **: P<0.01; ***: P<0.001 at each comparison with day 1. For statistics, see the Materials and Methods. Flies were maintained at 25°C.

**Figure S3.**

**
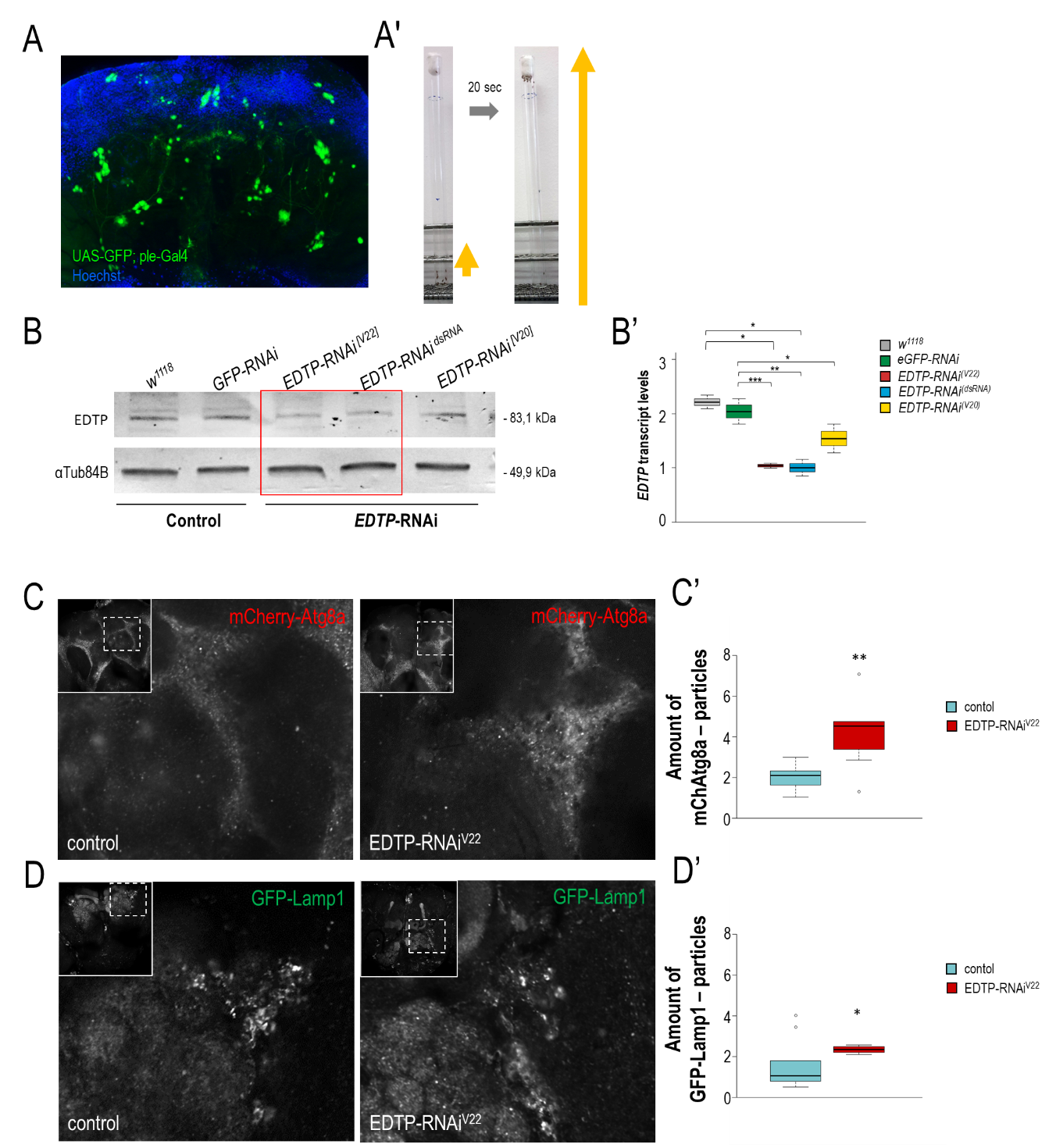
**

**Figure S3. *EDTP* downregulation in dopaminergic neurons.** (**A**) Expression of a *ple-Gal4* driver in dopaminergic neurons. The system restricts *EDTP* downregulation to dopaminergic neurons only (green). Hoechst staining (blue) indicates nuclei. (**A’**) Climbing assay used in this study**.** Animals were first collected at the bottom of a 25 cm long glass vial by tapping, then allowed to climb up on the wall (negative geotaxis). The number of animals reaching the top within 20 sec was determined. (**B**) Downregulation of *EDTP* by three different RNAi constructs. Western blot analysis showing that *V22* and *dsRNA* RNAi constructs (red frame) work effectively, so they were used further in this study. (**B’**) Quantification of *EDTP* transcript levels in brain samples isolated from staged adults. (**C-D’**) *EDTP* downregulation increases the amount of mCherry-Atg8a- and GFP-Lamp1-positive structures in animals at the stage of 21 days. Animals were maintained at 29°C. (**C’** and **D’**) Quantification of mCherry-Atg8a- and GFP-Lamp1-positive structures in brain samples. On the plot, the boxes represent the most typical 50% of the samples, the line indicates the median, upper and lower whiskers show remaining 25%-25% of the samples. Circles mark outliers. *: P<0.05; **: P<0.01; ***: P<0.001 at each comparison with day 1. For statistics, see the Materials and Methods.

**Figure S4.**


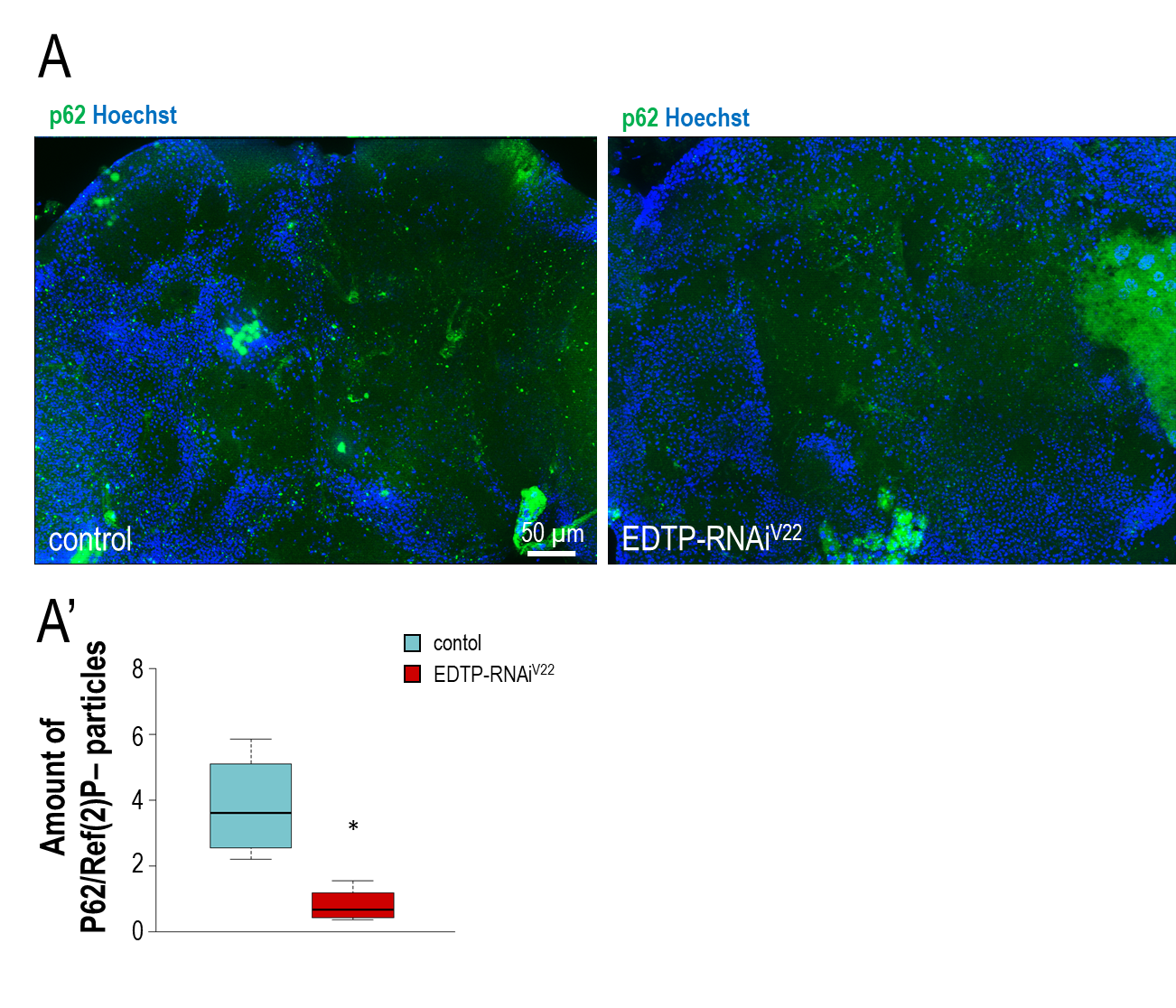


**Figure S4. *EDTP* downregulation lowers the amount of Ref(2)P-positive structures.** The amount of Ref(2)P-positive structures decreases in animals at the stage day 21 compared with control at the same stage. Animals were maintained at 29°C, an anti-Ref(2)P antibody was used for labelling. Hoechst staining (blue) indicates nuclei. Scale bars represent 50 µm. On the plot, the boxes represent the most typical 50% of the samples, the line indicates the median, upper and lower whiskers show remaining 25%-25% of the samples. Circles mark outliers. *: P<0.05; **: P<0.01; ***: P<0.001 at each comparison with day 1. For statistics, see the Materials and Methods.

**Figure S5.**

**
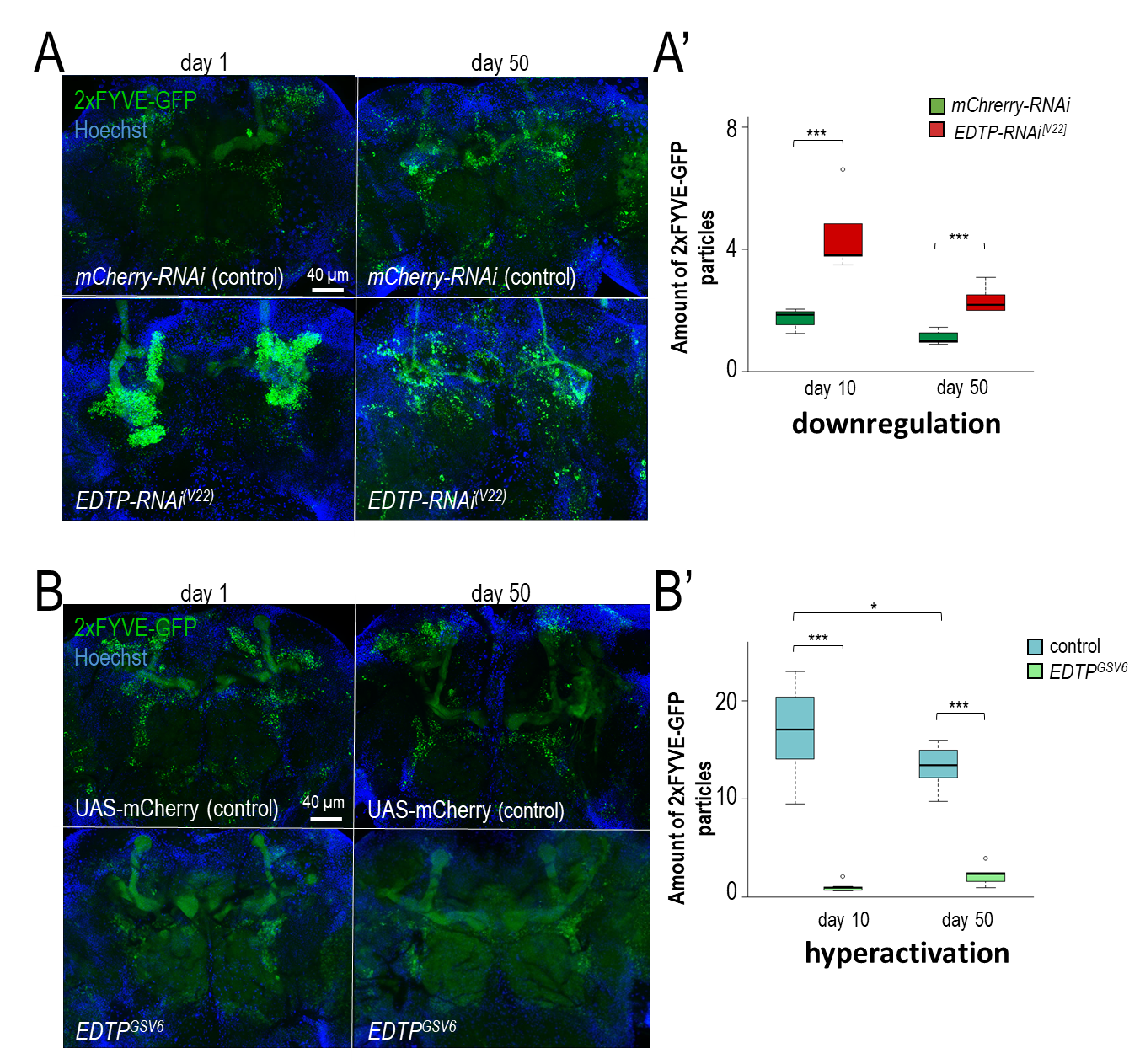
**

**Figure S5. EDTP activity influences PI3P accumulation.** (**A**) Fluorescent images displaying control (up) and *EDTP* downregulated (bottom) samples at the adult ages of day 10 (left) and 50 (right). (**A’**) Quantification of relative amounts of PI3P-positive structures**.** (**B**) Fluorescent images showing control (up) and EDTP-overexpressing (bottom) samples at the adult age of day 10 (left) and 50 (right). (**B’**) Quantification of relative PI3P area ratio**.** In panels A to B, RNAi constructs were driven by *App-Gal4,* green foci indicate 2xFYVE-GFP-labelled PI3P-positive structures, Hoechst staining (blue) indicates nuclei. Scale bars represent 40 µm. On the plot, the boxes represent the most typical 50% of the samples, the line indicates the median, upper and lower whiskers show remaining 25%-25% of the samples. Circles mark outliers. *: P<0.05; **: P<0.01; ***: P<0.001 at each comparison with day 1. For statistics, see the Materials and Methods.

**Figure S7.**

**
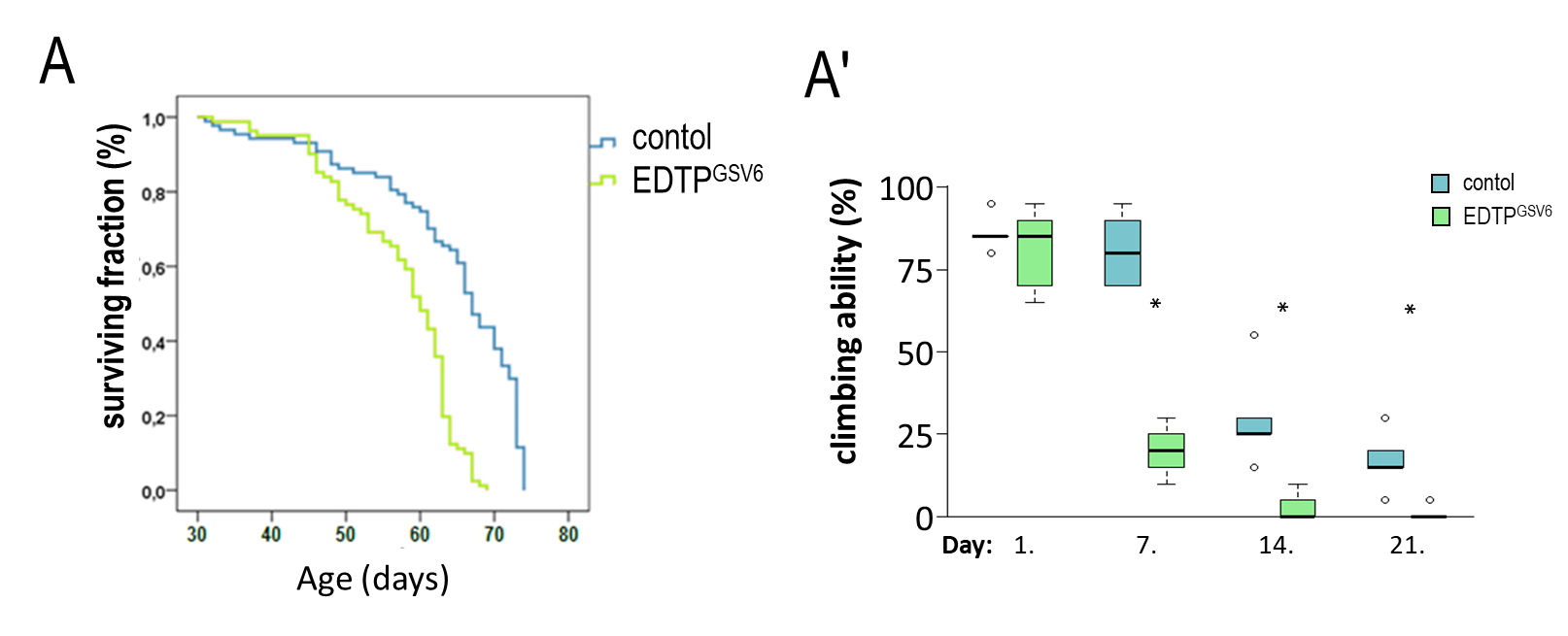
**

**Figure S7. EDTP hyperactivity limits lifespan and decreases climbing ability.**  (**A**) Animals over-expressing *EDTP* in the brain from the adult stage of 30th day live shorter than control. Kaplan-Meyer lifespan curves of control and EDTP-overexpressing (*EDTP^GSV6^*) animals. (**A’**) The ability of animals to climb up on the wall of a glass vial becomes decreased in response to EDTP hyperactivity in brain neurons. On the plot, the boxes represent the most typical 50% of the samples, the line indicates the median, upper and lower whiskers show remaining 25%-25% of the samples. Circles mark outliers. *: P<0.05; **: P<0.01; ***: P<0.001 at each comparison with day 1. For statistics, see the Materials and Methods.

**Figure S7.**

**
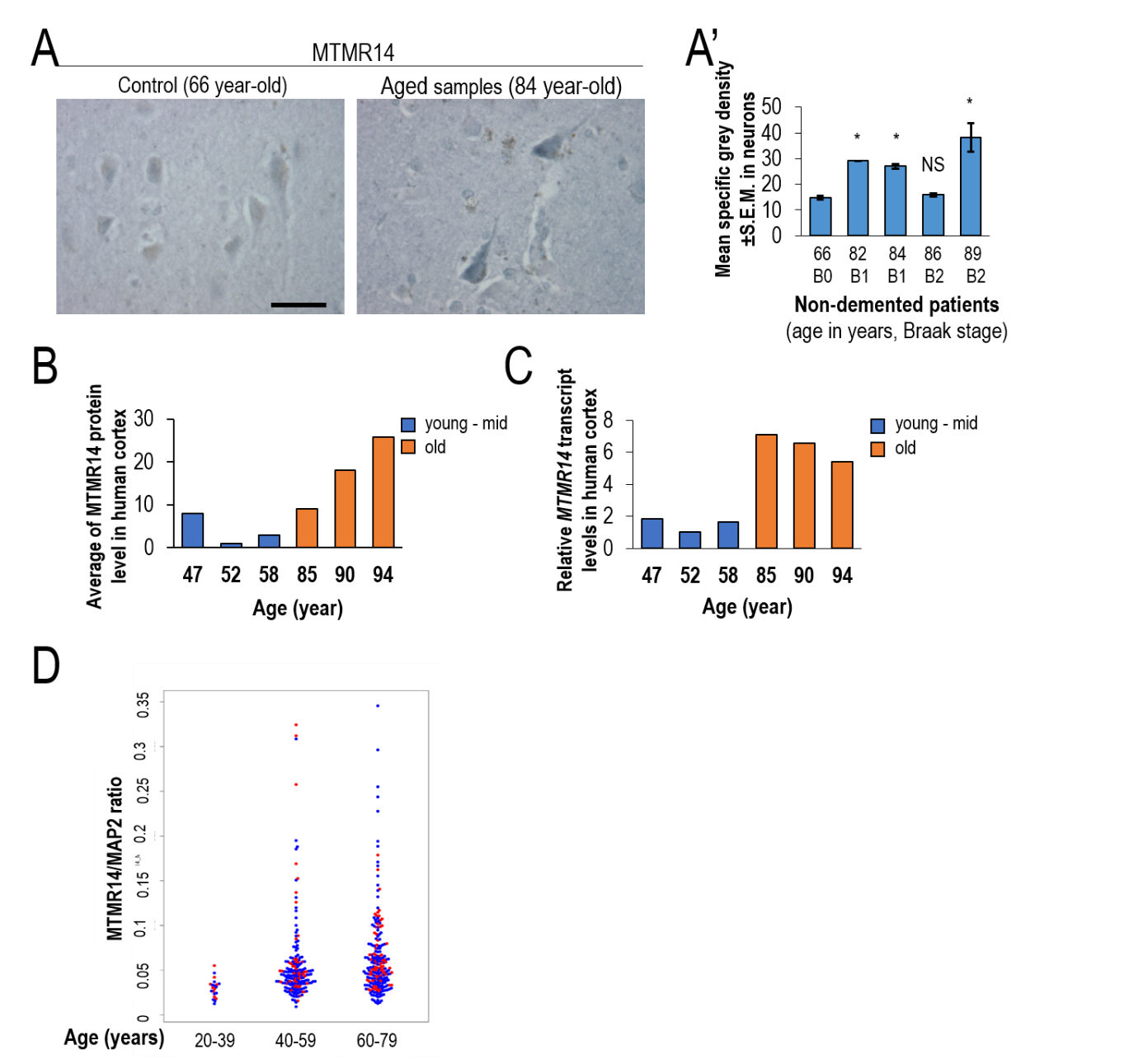
Figure S7. MTMR14 accumulates in brain neurons at higher levels at advanced ages compared with younger stages.** (**A**) Representative light microscopic immunohistochemical images from a 66-year-old male subject (left) and a 84-year-old male subject (right) demonstrate the accumulation of MTMR14 immunoreactivity as a function of age. Bar indicates 20 µm. (**A’**) Quantification of MTMR14 immunoreactivities. Bars represent specific mean density values ±S.E.M.), *: *P*< 0.001 (mean specific grey density values that are significantly increased over the value of the 66 year-old individual). For statistics, see Table S4. (**B**) MTMR14 protein levels increase during ageing in human cortex. GAPDH was used as an internal control (western blot). (**C**) *MTMR14* transcript levels in human cortex increases with age (qPCR analysis). For statistics, see Table S5 and Materials and Methods. (**D**) The ratio of *MTMR14*:*MAP2* transcript levels was determined. Data were extracted from GTExPORTAL version 7 (<https://gtexportal.org/home/>). Input of 408 RNAseq samples (combined frontal cortex, cortex and cingulate cortex) was grouped into 3 age groups (20-39, 40-59 and 60-79 years). MTMR14 expression levels were normalized to neuronal content, based on *MAP2* expression. Created beeswarm plots show male (blue) and females (red) dots. Groups and mean RNAseq data: 20-39 years, 0.0298; 40-59 years, 0.0573; 60-79 years, 0.065. Tukey was performed to find significant changes (Tukey multiple comparisons of means, 95% family-wise confidence level). Differences between groups: I. 40-59 years – 20-39 years, 0.027476363 (diff), 0.001779765 (lwr), 0.05317296 (upr), *P*<0.0328198; II. 60-79 years – 20-39 years 0.035202536 (diff), 0.009747328 (lwr), 0.06065774 (upr), *P*<0.0035359; III. 60-79 years – 40-59 years 0.007726173 (diff), 0.003638182 (lwr), 0.01909053 (upr), *P*<0.2471079.


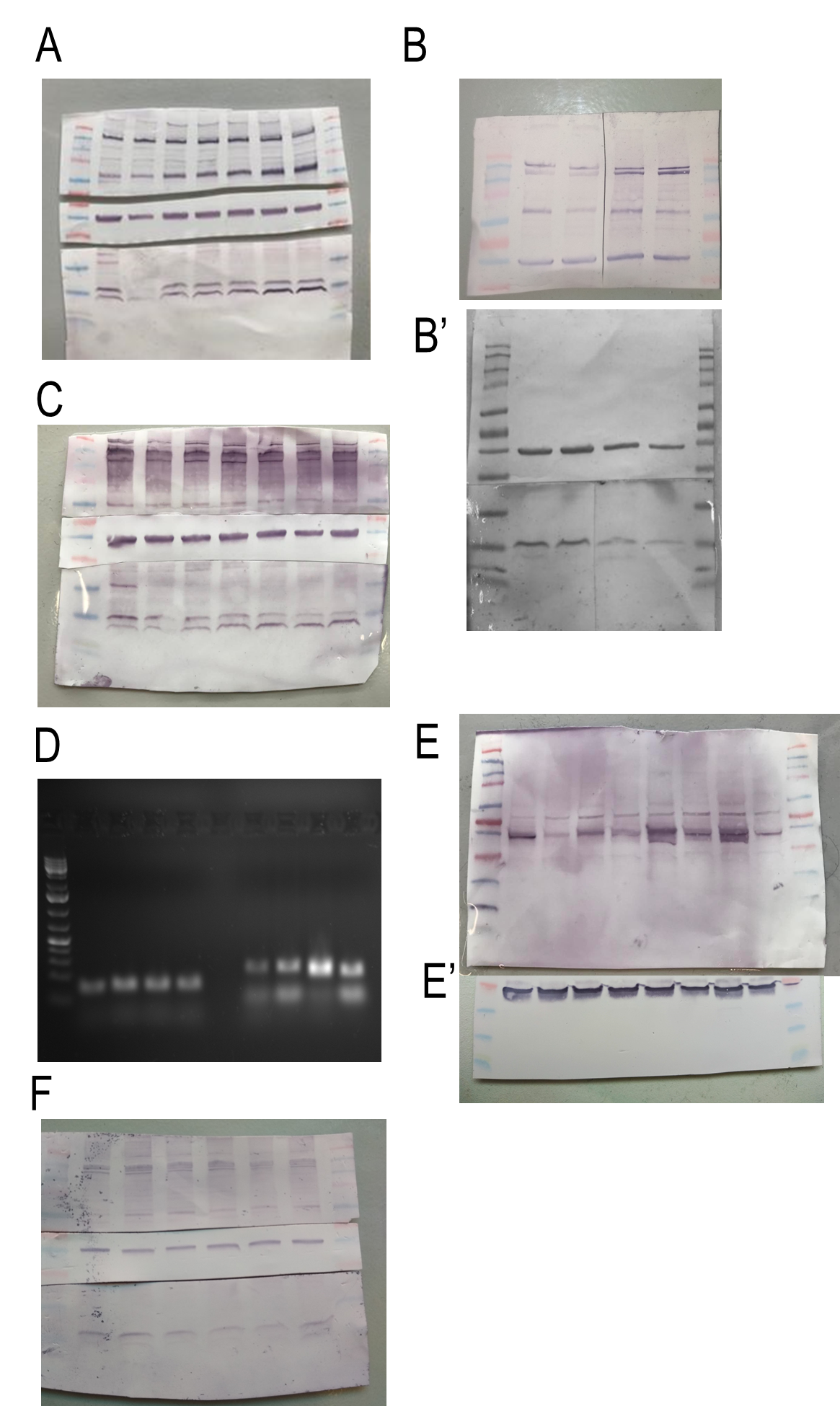


**Figure S8. Original images of western blot and PCR results.** The following images represent the original images of the western blot and PCR results: **A)** Figure 1 C, **B)** and **B’)** Figure 4 C, **D)** Figure 4 D and **E** and **E’** Figure 6 C, **F)** Figure S. 3. After blotting, the membranes were cut into several parts because different proteins were labelled with different antibodies.

**Supplementary Tables**

**Table S1. Statistics for lifespan data.**

| **Genotype (crossing)** | **Number of flies examined** | **Number of repeats (vials)** | **Mean lifespan (days)** | **±S.E.M. (days)** | **Log Rang value** | **Mean survival**  **Mann-Whitney U-test *P* value** |
| --- | --- | --- | --- | --- | --- | --- |
| for **Figure 4C** to **C’** | | | | | | |
| ple-Gal4 x *eGFP*-RNAi (V22) | 85 | 4 | 51.835 | 1.974 | vs. *w^1118^* *P*=0.018 |  |
| ple-Gal4 x *EDTP*-RNAi (V22) | 176 | 5 | 55.312 | 1.389 | vs. *w^1118^* *P*=0.0001  vs. *eGFP-RNAi P*=0.017 | vs. *w^1118^* *P*=0.0007  vs. *eGFP-RNAi P*=0.0457 |
| for **Figure 4D** to **D’** | | | | | | |
| ple-Gal4 x *eGFP*-RNAi (V22) | 185 | 5 | 48.346 | 1.454 | vs. *w^1118^* *P*=0.018 |  |
| ple-Gal4 x *EDTP*-RNAi (V22) | 179 | 5 | 50.41 | 1.561 | vs. *w^1118^* *P*=0.002  vs. *eGFP-RNAi P*=0.0001 | vs. *w^1118^* *P*=0.0001  vs. *eGFP-RNAi P*=0.0489 |
| ple-Gal4 x *EDTP*-RNAi (dsRNA) | 134 | 4 | 51.441 | 1.263 | vs. *w^1118^* *P*=0.015  vs. *eGFP-RNAi P*=0.001  vs. *EDTP-RNAi(V22) P*=0.241 | vs. *w^1118^* *P*=0.0003  vs. *eGFP-RNAi P*=0.5104  vs. *EDTP-RNAi(V22) P*=0.62 |

**Table S2. Clinical and vital characteristics of human subjects studied for SQSTM1/p62 immunoreactivity.**

| **Subjects** | **Age (year)** | **Post-mortem delay (min)** | **Sex** | **Braak stage** |
| --- | --- | --- | --- | --- |
| **Non-demented aged persons** | **77 ± 8** | **653 ± 478** |  |  |
| 95/307 | 73 | 330 | F | 1 |
| 95/339 | 81 | 1335 | F | 1 |
| 96/030 | 68 | 630 | F | 2 |
| 96/049 | 86 | 315 | F | 1 |

Tissue samples were obtained from the Netherlands Brain Bank (Project 598/2009), Netherlands Institute for Neuroscience, Amsterdam. Averaged data are expressed as mean ± S.D. M: male, F: female.

**Table S3. MTMR14 Immunoreactivity-specific optical intensity values in human cortical tissues.**

| **Sample** | counted cells | cortical tissues |
| --- | --- | --- |
| SKO20/Br38 | 33 | 22.02 ± 8.77 |
| SKO7/Br38 | 31 | 18.71 ± 6.68 |
| SKO19/Br38 | 32 | 19.16 ± 5.43 |
| SKO11/Br38 | 30 | 22.53 ± 8.31 |
| SKO16/Br38 | 30 | 19.12 ± 5.97 |
| SKO18/Br38 | 31 | 25.30 ± 8.17 |
| statistical analysis (merged) form results of upper samples | | |
| YOUNG | 96 | 20.01 ± 7.15 |
| OLD | 91 | 22.35 ± 7.91* |

Data are expressed as means of anti-MTMR14 immunofluorescence relative intensity values ±S.D. *: P=0.034 (independent samples t-test).

**Table S4. Clinical characteristics of human subjects studied for MTMR14 immunoreactivity.**

| **Subjects** | **Age (year)** | **Post-mortem delay (hour)** | **Sex** | **Category** |
| --- | --- | --- | --- | --- |
| SKO20/Br38 | 27 | 4-5 hours | M | YOUNG |
| SKO7/Br38 | 55 | 3-4 hours | M | YOUNG |
| SKO19/Br83 | 61 | 3 hours | F | YOUNG |
| SKO11/Br38 | 72 | 3 hours | M | OLD |
| SKO16/Br38 | 77 | 2-3 hours | M | OLD |
| SKO18/Br38 | 85 | 4-5 hours | M | OLD |

**Table S5. Clinical characteristics of human subjects studied for MTMR14 fluorescent microscopy, qPCR and Western blot analyses.**

| **Subjects** | **Age (year)** | **Post-mortem delay (hour)** | **Sex** | **Category** |
| --- | --- | --- | --- | --- |
| Or F120 right | 47 | 6 hours | M | MID |
| Or E965 right | 52 | 8 hours | M | MID |
| Or F216 right | 58 | 6 hours | F | MID |
| Or E692 left | 85 | 8 hours | F | OLD |
| Or D259 left | 90 | 4-5 hours | M | OLD |
| Y E249 left | 94 | 4 hours | M | OLD |

**Table S6. Individual dog samples for determining age-associated *MTMR14* expression levels in the prefrontal cortex.** For Figure 6C, transcript levels were determined by qRT-PCR.

| **Table of individual dogs** | | |
| --- | --- | --- |
| **Breed** | **Age (years)** | **Category** |
| golden retriever | 17 | OLD |
| small münsterlander | 17 | OLD |
| border collie | 14 | OLD |
| labrador | 14 | OLD |
| labrador | 14 | OLD |
| labrador | 13 | OLD |
| beagle | 3 | YOUNG |
| beagle | 3 | YOUNG |
| beagle | 3 | YOUNG |
| beagle | 3 | YOUNG |
| beagle | 3 | YOUNG |
| beagle | 3 | YOUNG |
| beagle | 3 | YOUNG |
| boxer | 1 | YOUNG |
| border collie | 1 | YOUNG |
